# Heterotypic transcriptional condensates formed by prion-like paralogous proteins canalize flowering transition in tomato

**DOI:** 10.1186/s13059-022-02646-6

**Published:** 2022-03-14

**Authors:** Xiaozhen Huang, Nan Xiao, Yupan Zou, Yue Xie, Lingli Tang, Yueqin Zhang, Yuan Yu, Yiting Li, Cao Xu

**Affiliations:** 1 State Key Laboratory of Plant Genomics, National Center for Plant Gene Research (Beijing), Institute of Genetics and Developmental Biology, The Innovative Academy of Seed Design, Chinese Academy of Sciences, Beijing, China; 2 CAS-JIC Centre of Excellence for Plant and Microbial Science (CEPAMS), Institute of Genetics and Developmental Biology, Chinese Academy of Sciences, Beijing, China; 3 University of Chinese Academy of Sciences, Beijing, China; 4 College of Coastal Agricultural Sciences Guangdong Ocean University, Zhanjiang, China

**Keywords:** Paralogs, Gene duplication, Genetic redundancy, Flowering transition, Phase separation, Transcriptional condensates, Developmental robustness

## Abstract

**Background:**

Paralogs that arise from gene duplications during genome evolution enable genetic redundancy and phenotypic robustness. Variation in the coding or regulatory sequence of paralogous transcriptional regulators diversifies their functions and relationships, which provides developmental robustness against genetic or environmental perturbation. The fate transition of plant shoot stem cells for flowering and reproductive success requires a robust transcriptional control. However, how paralogs function and interact to achieve such robustness is unknown.

**Results:**

Here, we explore the genetic relationship and protein behavior of ALOG family transcriptional factors with diverse transcriptional abundance in shoot meristems. A mutant spectrum covers single and higher-order mutant combinations of five ALOG paralogs and creates a continuum of flowering transition defects, showing gradually enhanced precocious flowering, along with inflorescence simplification from wild-type-like to progressively fewer flowers until solitary flower with sterile floral organs. Therefore, these paralogs play unequal roles and act together to achieve a robust genetic canalization. All five proteins contain prion-like intrinsically disordered regions (IDRs) and undergo phase separation. Accumulated mutations following gene duplications lead to IDR variations among ALOG paralogs, resulting in divergent phase separation and transcriptional regulation capabilities. Remarkably, they retain the ancestral abilities to assemble into a heterotypic condensate that prevents precocious activation of the floral identity gene *ANANTHA*.

**Conclusions:**

Our study reveals a novel genetic canalization mechanism enabled by heterotypic transcriptional condensates formed by paralogous protein interactions and phase separation, uncovering the molecular link between gene duplication caused IDR variation and robust transcriptional control of stem cell fate transition.

**Supplementary Information:**

The online version contains supplementary material available at 10.1186/s13059-022-02646-6.

## Background

Within a population, major phenotypes are relatively stable and independent of genetic and environmental variabilities. This stability is maintained by a genetic process called canalization, the source of robustness of a biological system [1, 2]. Flowering plants have developed genetic circuitry that buffers against genetic and/or abiotic perturbations to avoid drastic morphological consequences. This type of canalization mechanisms not only maintain the robustness of core developmental programs to preserve phenotypes shaped by long history of natural selection, but also retain the flexibility for phenotypic innovation [3, 4]. Not surprisingly, in flowering plants, flowering time and inflorescence architecture, which convey reproductive success, are heavily canalized and show great robustness. Functional redundancy following gene duplications and gene family expansion are commonly hypothesized to underlie phenotypic robustness [3]. However, exactly how phenotypic robustness is maintained in the context of duplicated genes’ fates is rarely studied and illustrated in plants. A major obstacle to study developmental robustness is the limited number of mutants available for genes in a large family, either by fortuitous natural mutations or artificial mutagenesis. Before dissecting a specific developmental program, a comprehensive view of the number and roles of the players involved is a prerequisite, which in a lot of cases is impeded by the lack of genomic and evolutionary analyses. This sometimes leads to mischaracterization of genetic components due to cryptic phenotypes masked by canalization in certain mutants.

Inflorescences develop from a group of pluripotent stem cells called shoot apical meristems (SAMs), whose fate are determined by balancing cell proliferation for maintaining stem cell population and differentiation for organogenesis [5, 6]. Upon perception and integration of endogenous and environmental cues, SAMs experience a gradual transition process from a vegetative state (vegetative meristem, VM) into a reproductive stage (floral meristem, FM) called meristem maturation [5, 7]. The programed meristem maturation ensures a timely transition to flowering and flower formation in appropriate organization and quantity, which directly impacts plant reproductive success and crop yield [5, 8]. Before reaching this crucial transition, SAM at vegetative stage is not ready to respond to endogenous or environmental signals. Therefore, a program preventing precocious maturation is vital to maintain an adequate stem cell population.

A repressing program that maintains meristem at a vegetative state is defined by a tomato ALOG (*Arabidopsis* LSH1 and *Oryza* G1) transcription factor, TERMINATING FLOWER (TMF) [9]. TMF harbors a conserved ALOG domain featured by a putative DNA-binding domain derived from the XerC/D-like recombinases of a novel class of retrotransposons [10, 11]. Loss of *TMF* in tomato leads to much faster flowering and conversion of a multiple-flowered primary inflorescence into a single flower. These effects are caused by a precocious activation of a F-box floral identity gene *ANANTHA* (*AN*; homolog of *Arabidopsis UFO* and petunia *DOT*), loss of which in tomato only produce overproliferated axillary inflorescence meristems and never form normal inflorescences and flowers, making a meristem mostly at a vegetative stage acquire floral identity [9, 12–15]. Surprisingly, inflorescences developed from side shoots of *tmf* mutant are normal compound inflorescences, suggesting that to certain extent, *TMF* paralogs in ALOG family or other players may have canalized inflorescence architecture regulation centered around *TMF*. Indeed, the tomato ALOG family includes twelve members called TMF FAMILY MEMBERs (TFAMs). Unlike *tmf*, the *tfam1* or *tfam2* mutant shows normal flowering time with only slightly increased occurrence of inflorescene branching and vegetative reversion [10]. Notably, *tfam1* exhibits defects in stamen development and floral abscission [10], suggesting potential functional divergence and subfunctionalization of ALOG paralogs. Studies in *Arabidopsis* and other nightshades (Solanaceae) also reported important roles of ALOG genes in floral organ specification, leaf development and light signaling [16–19]. In rice and wheat, several ALOG family members have been found to regulate lemma and hull specification, spikelet development, panicle branching, and grain size [20–23]. Our recent study in tomato has discovered that developmentally produced reactive oxygen species (ROS) in SAMs induce phase separation of TMF to form transcriptional condensates, which repress expression of the floral identity gene *ANANTHA* to regulate flowering transition [24]. Loss of *Marchantia polymorpha LATERAL ORGAN SUPRESSOR1* (*MpLOS1*), a *TMF* ortholog in liverwort, causes mis-specified identity of lateral organogenesis and defects in apical meristem maintenance, suggesting its essential role in convergent evolution of lateral organogenesis [25]. Therefore, ALOG proteins represent a conserved transcription factor family with vital functions in meristem activity tracing back to early land plants. Current redundancy in vegetative meristem maintenance by TFAMs in tomato could be a robust biological system created by a gene family expansion and various evolutionary fates of its members. However, the molecular mechanism of the functional redundancy, coordination and transcriptional regulation is still unknown.

Plant meristem maturation is controlled by precise on/off switches of gene repression and activation, which requires integration of endogenous and environmental signals into fine-tuned spatial/temporal gene expression in a stable micro-environment. Biomolecular condensates formed by protein phase separation have been demonstrated as a new strategy for many cellular functions in yeast and animal systems, including cell polarity establishment and maintenance, cell signaling, cell and organ development, cell survival, and aging [26]. With few studies, the mechanisms of protein phase separation and the function of the derived biomolecular condensates in plant development are still largely uninvestigated [24, 27–29]. Here, we take advantage of CRISPR/Cas9 technology and genetic stacking to generate a full spectrum of single, double, triple, quadruple mutants of TMF and TFAMs. We elucidate how TFAM proteins work together via heterotypic condensation to achieve a robust transcriptional control of stem cell fate transition in tomato.

## Results

### Unequal genetic redundancy of ALOG paralogs underlies tomato flowering and inflorescence architecture

**Fig. 1:**
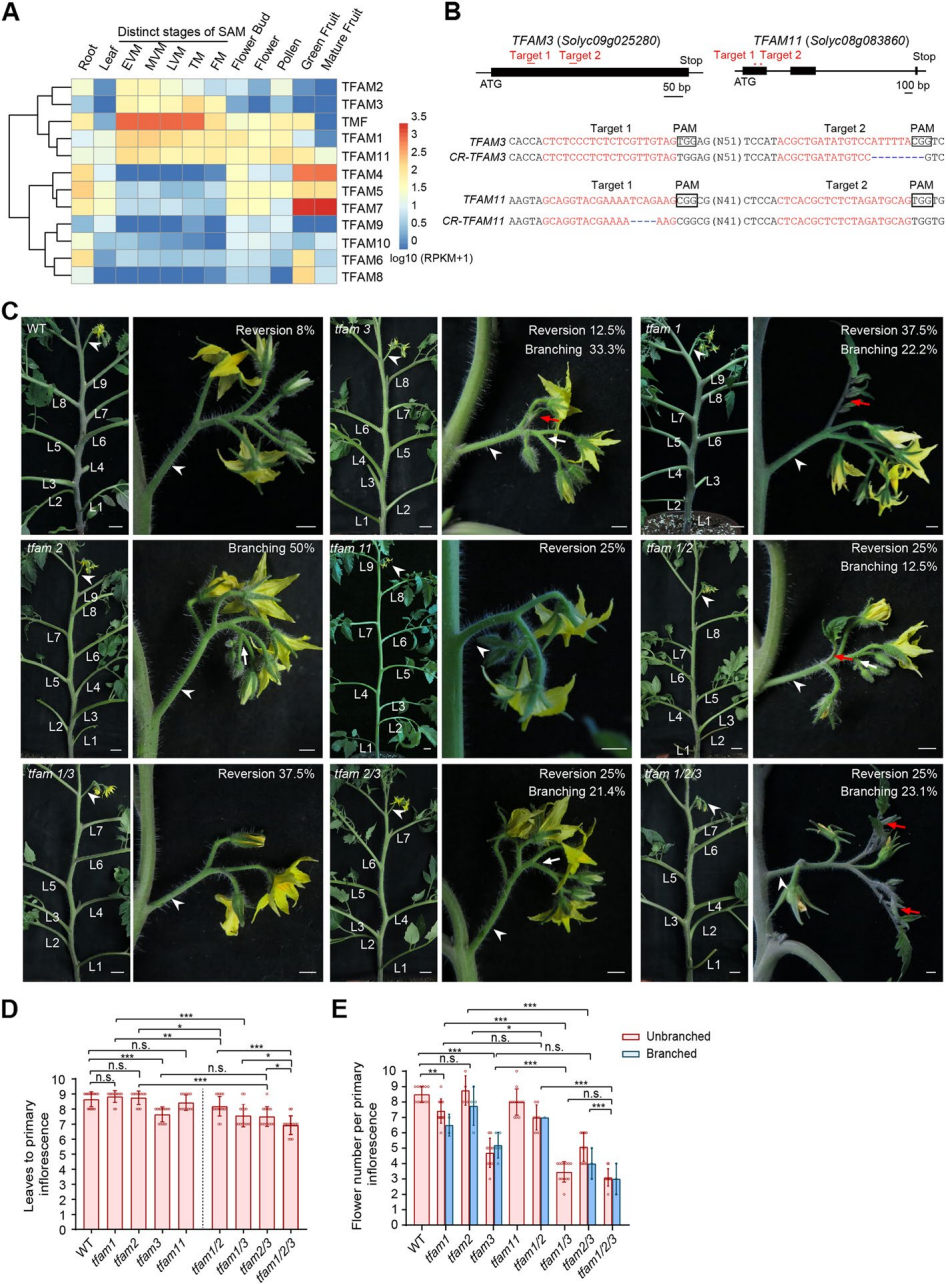
Identification of ALOG paralogs required for flowering transition. **A** Phylogenetic relationship and expression dynamics of ALOG family members in tomato. EVM, early vegetative meristem; MVM, middle vegetative meristem; LVM, late vegetative meristem; TM, transitional meristem; FM, floral meristem. **B** Schematic (upper) indicating sgRNAs (red lines) and allelic information (bottom) for *TFAM3*, *TFAM11*, respectively. **C** Representative shoot and typical primary inflorescences from WT and *tfam* single, double and triple mutants. White arrowheads indicate inflorescences, red arrows indicate vegetative reversions, and white arrows indicate branching events on inflorescences. L, leaf. Scale bars, 2 cm for plants; 0.5 cm for inflorescences. **D**, **E** Statistics of flowering time (**D**) and flower number per inflorescence (**E**) for WT and *tfam* single, double, and triple mutants. The flower numbers were quantified from branched and unbranched inflorescences separately. Data are means ± SD (*n* = 17, 12, 12, 14, 14, 16, 14, 14, 14, for B; *n* = 12, 14, 8, 15, 12, 8, 13, 14, 13 for C, *P < 0.05, **P < 0.01, ***P < 0.001, Student *t*-test)

The fact that TFAM family members not only retain partial functions in flowering and inflorescence complexity but also subfunctionalize in other developmental processes makes the gene family ideal for exploring paralogous relationships. To this end, we firstly examined the phylogeny relationship and expression pattern of TFAMs in different organs and meristems at various developmental stages. The twelve ALOG genes can be divided into two major clusters, one of them includes five members, *TMF* and *TFAM1*, *2*, *3*, *11*, expressing at distinct developmental stages of SAM maturation, albeit with different transcription levels (Fig. 1A). Among them, *TMF* shows the most abundant expression. Given that TFAM3 and TFAM11 are the only two unknown paralogs in this cluster, we then used CRISPR/Cas9 system to knock out them individually to analyze the phenotypic consequences (Fig. 1B). Disruption of SAM maturation program often results in change of flowering time, extra branching, vegetative reversion, and flower number reduction on inflorescences [5, 10]. The *tfam3* null mutant flowers about one leaf earlier and produces fewer flowers on the primary inflorescence compared with wild type (Fig. 1C-E), resembling mutant phenotypes of *TMF* weak alleles [9, 24]. Moreover, about 13% of the primary inflorescences of *tfam3* show vegetative reversion, represented by outgrowth of leaves on the primary inflorescence (Fig. 1C). The *tfam3* mutant frequently undergoes a single branching event on each inflorescence (Fig. 1A). These phenotypes remind us the inflorescence development defects of *tfam1* and *tfam2* mutants. As previously reported and shown here, flowering time in terms of leaves before primary inflorescence is unaffected in *tfam1* and *tfam2* single mutants (Fig. 1C, D). Instead, *tfam1* shows reduced flower production but highly frequent vegetative reversion on inflorescences, and *tfam2* develops inflorescences often containing single branching event (Fig. 1C, E) [10]. Interestingly, the *tfam11* null mutant resembles wild-type plants in contrast to the modest-to-strong phenotypes of *tfam1*, *2*, *3* and *tmf* mutants, suggesting the existence of unequal genetic redundancy. Though *TFAM11* shows comparable, even higher transcriptional level than *TFAM1*, *2* and *3* in the SAM (Fig. 1C, E), its null mutant is indistinguishable from wild-type, indicating that *TFAM11* is dispensable in this process, and the unequal redundancy among these TFAMs is not merely due to transcription level. Together, these findings suggest that *TFAM3* is a novel regulator that represses flowering transition and promotes inflorescence complexity. The observed phenotypic similarities and variance in the degree of defects of the four *tfam* mutants suggest that their functions could be unequally redundant.

### Genetic stacking of *tfam* mutants produces a phenotypic continuum of flowering and inflorescence complexity

Regarding the varying phenotypic severity ranging from wild-type-like of *tfam11* to weak-to-modest of *tfam1*, *2* and *3*, we generated various combinations of higher-order mutants by genetic crosses between *tfam1*, *2* and *3* to explore how they coordinate in synchronizing flowering transition and influence inflorescence complexity. Loss of either *TFAM1* or *TFAM2* in *tfam3* mutant background enhances its early flowering phenotypes: one leaf earlier than *tfam3* single mutant, about two leaves earlier than wild type plants (Fig. 1C, D). The *tfam1/2* flowered faster than either single mutant by about one leaf, showing weaker early-flowering phenotype than *tfam1/3* and *tfam2/3* (Fig. 1C, D). In addition to enhanced flowering time phenotype, the *tfam* double mutants showed a range of modifications in inflorescence architecture. In the most dramatic case, *tfam1/3* inflorescences produce less than half of flowers compared to wild-type plants, and about 38% inflorescences show vegetative reversion, similar to *tfam1* but stronger than that of *tfam3* (Fig. 1C–E). In contrast, *tfam2/3* and *tfam1/2* only displayed a slight change of vegetative reversion frequency and branching compared to single mutants (Fig. 1C–E). Strikingly, compared to *tfam* double mutants, *tfam1/2/3* triple mutant showed similar frequency of vegetative reversion and branching, but it flowered even faster, and produced only three flowers on inflorescences (Fig. 1C–E). The progressive enhancement of precocious flowering and inflorescence “vegetativeness” displayed by this complete series of *tfam* mutants illustrates their inseparable relationship and synergistic effects in modulating flowering time and inflorescence architecture.

**Fig. 2:**
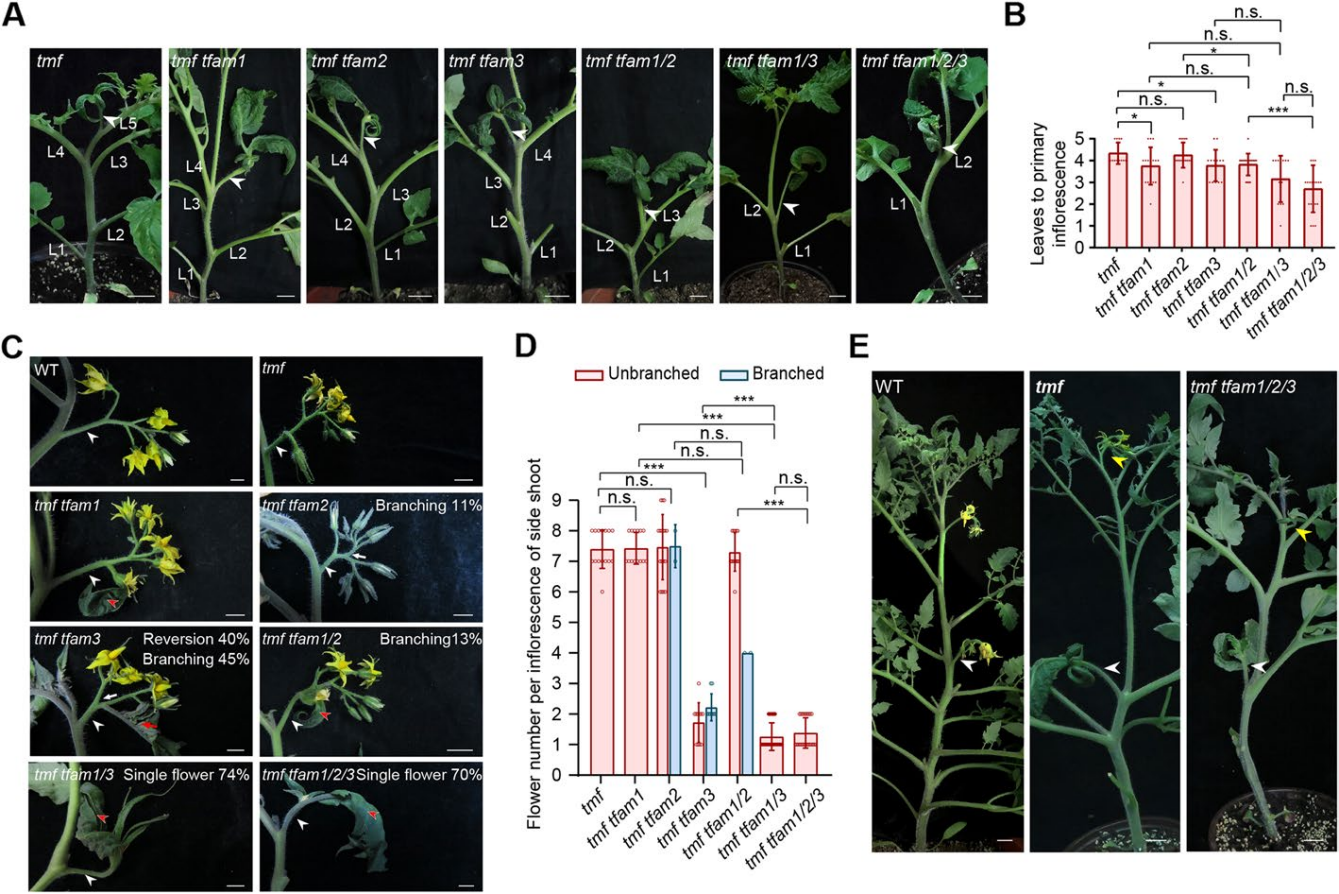
Genetic interactions between *TMF* and *TFAM* genes. **A**, **B** Representative shoots with primary inflorescence (**A**) and quantification of flowering time (**B**) for *tmf* single mutant and higher-order mutants of *tmf* and *tfams*. White arrowheads indicate single-flowered primary inflorescences. Data are means ± SD (*n* = 12, 16, 16, 13, 22, 13, 20, *P < 0.05, **P < 0.01, ***P < 0.001, Student *t*-test). L, leaf. Scale bars, 2 cm. **C**, **D** Images of inflorescence (**C**) and quantification of flower number per inflorescence (**D**) from side shoots of various mutant combinations. Red arrowheads indicate leaf-like sepal, and white arrowheads indicate inflorescences. Data are means ± SD (*n* = 15, 14, 19, 20, 15, 22, 25, ***P < 0.001, Student *t*-test). Scale bars, 1 cm. **E** Representative shoot with two successive inflorescences for WT, *tmf* single mutant and *tmf tfam1/2/3* quadruple mutant. White arrowheads and yellow arrowheads indicate primary inflorescences and side-shoot inflorescences, respectively. Scale bars, 2 cm

The early-flowering and simplified-inflorescence phenotypes of various *tfam* mutants are similar, albeit weaker, compared to the original *tmf* null mutant, consistent with the higher expression level of *TMF* than *TFAM1*, *2* and *3* in vegetative meristems. We therefore hypothesize that TMF might function as a dominant player to coordinate the concerted effects of three TFAMs. To test this, we crossed all single and multiple *tfam* mutants with *tmf* to create a series of *tmf tfam* mutant combinations. Among the double mutants, *tmf tfam1* and *tmf tfam3* showed the most significant enhancement of early-flowering comparing to *tmf* (Fig. 2A, B). In contrast, there is no significant difference in flowering between *tmf tfam2* and *tmf* (Fig. 2A, B). More prominent enhancement of early-flowering occurs in triple mutants. The *tmf tfam1/2* and *tmf tfam1/3* flowered earlier than *tmf* single mutant by one and two leaves, respectively (Fig. 2A, B). Strikingly, the *tmf tfam1/2/3* quadruple mutant flowered extremely early after producing only two leaves and developed single-flowered inflorescences (Fig. 2A, B). In addition, the *tmf* single-flower phenotype showed about 80% penetrance under our growth conditions. Neither introduction of *TFAM1*, *2* mutation individually nor simultaneously significantly improves the penetrance. However, the *tmf tfam1/2/3* quadruple mutant showed almost 100% penetrance for the single-flower phenotype (Additional file 1: Fig. S1). *tmf* is single-flowered, and this flower often develops leaf-like sepals [9, 10]. Interestingly, introducing more mutations of *TFAM* genes into *tmf* background causes sepals of the solitary flower to show more leafy characteristics (Fig. 2A). These findings suggest that *TMF* requires *TFAM*s to achieve the precise control of flowering transition, among which *TFAM1* and *TFAM3* contribute much more than *TFAM2*.

While the primary inflorescence of *tmf* is single-flowered, inflorescences that develop from side shoots are unaffected [9], suggesting existence of redundant factors. We then examined the inflorescences from side shoots of various higher-order mutants of *tmf* and *tfams*. Quantification of flower number per inflorescence from side shoots showed no significant difference between *tmf*, *tmf tfam1*, *tmf tfam2*, *tmf tfam1/2* mutants and wild type plants (Fig. 2C, D), however, *tmf tfam1* and *tmf tfam1/2* displayed leaf-like sepals at the first flower on the side shoot inflorescences (Fig. 2C). Notably, side shoot inflorescences of *tmf tfam3* double mutant almost always produce only two flowers, and most of the inflorescences showed vegetative reversion and branching (Fig. 2C, D). Significantly, approximately 74% of side shoot inflorescences from *tmf tfam1/3* are single-flowered with extremely leaf-like sepals (Fig. 2C, D), which never appears in *tfam1/2/3* triple mutants (Fig. 1C, E). Interestingly, the side shoot inflorescences of *tmf tfam1/2/3* are mostly undistinguished from *tmf tfam1/3* (Fig. 2C-E). Together, these results suggest that *TMF*, *TFAM1* and *TFAM3* work in finely controlled coordination in flower production on both primary and axillary shoots with potential subfunctionalization shown by the division of labor among TFAMs.

### TMF and TFAMs synergistically repress SAM maturation

**Fig. 3:**
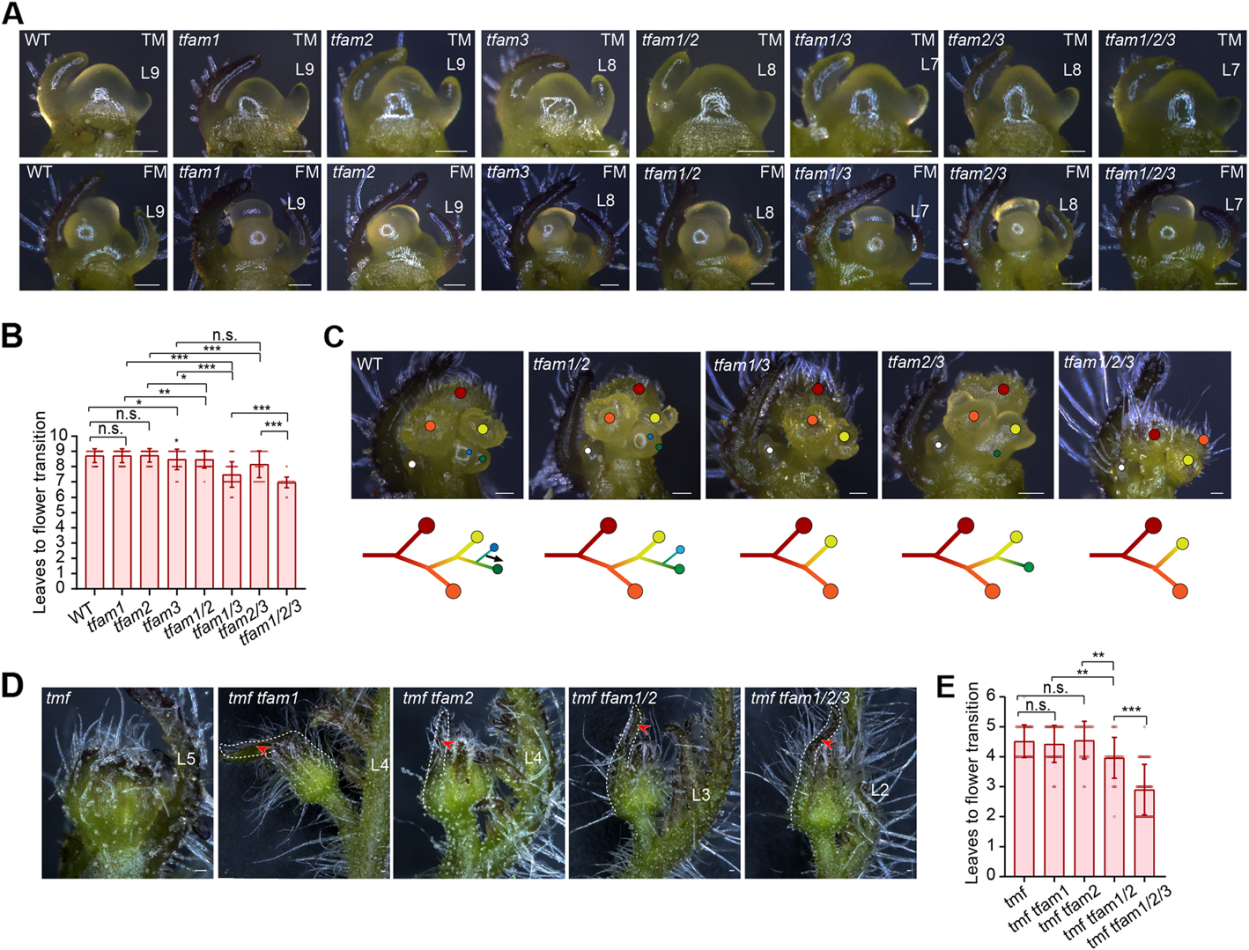
Developmental basis of shoot apical meristem maturation in single and higher-order mutants of *TFAMs*. **A**, **B** Stereoscope images of meristems (**A**) and quantification data of leave primordium production for flower transition (**B**) from WT and *tfam* single, double, and triple mutants. Data are means ± SD (*n* = 69, 46, 46, 42, 59, 54, 39, 73, *P < 0.05, **P < 0.01, ***P < 0.001, Student *t*-test). Scale bars, 100 μm. L, leaf. **C** Young inflorescences (upper) and diagrams (bottom) of WT and *tfam* mutants. Colored dots indicate terminated FM (red, orange and yellow dots) and initiated sympodial inflorescence meristem (SIM) (blue and green dots). White dots indicate the first sympodial shoot meristem (SYM). The black arrow indicates continued SIM reiteration. Scale bars, 100 μm. **D**, **E** Stereoscope images of floral meristem (**D**) and quantification of leaf production for flower transition (**E**) from *tmf* and *tfam* mutants. Data are means ± SD (*n* = 53, 42, 29, 29, 62, **P < 0.01, ***P < 0.001, Student *t*-test). Red arrowheads indicating the leaf sepal at floral meristem stage. Scale bars, 100 μm. L, leaf

To explore the developmental basis of the flowering and inflorescence defects in various single and high-order mutants, we dissected and compared the SAM at reproductive stages. SAMs of the *tfam* single and higher mutants are morphologically indistinguishable at the transitional meristem (TM) stage (Fig. 3A). However, the maturation rate of the SAMs, indicated by the number of leaf primordium produced before vegetative meristems transitioning into floral meristems, varied in different mutants. Although neither *tfam1* nor *tfam2* showed modified maturation rate, *tfam3*, *tfam1/2*, and *tfam2/3* transitioned faster than wild type by about one leaf primordium (Fig. 3A, B). *tfam1/3* and *tfam1/2/3* exhibited the fastest maturation rate, producing about two leaf primordium fewer than wild type at floral transition stage (Fig. 3A, B). The faster maturation gave rise to the early-flowering phenotypes in those mutants. The inflorescence complexity can be measured by the number of AIM initiated at young inflorescence stage [10]. *tfam1/2* showed slightly slower initiation of AIMs, but *tfam1/3*, *tfam2/3*, and *tfam1/2/3* initiated significantly fewer AIMs than wild type (Fig. 3C). The precocious termination of AIM initiation translates into simplified inflorescences in these *tfam* mutants. Examining young inflorescences of various combinations of *tmf* and *tfam* mutants, extremely simplified inflorescences can be observed, featuring single flowers with leaf-like sepals (Fig. 3D). The progressive enhancement of flowering and inflorescence defects with increasing mutated TFAM genes are reflected by the number of leaf primordium production in SAM before floral transition and the size of leaf-like sepals in mature inflorescences (Fig. 3D, E). Taken together, these results indicated that TMF and TFAMs act together to fulfill the genetic canalization that ensures robustness of vegetative meristem maintenance for flowering transition.

### TFAM proteins form biomolecular condensates in the nucleus of tomato cells

**Fig. 4:**
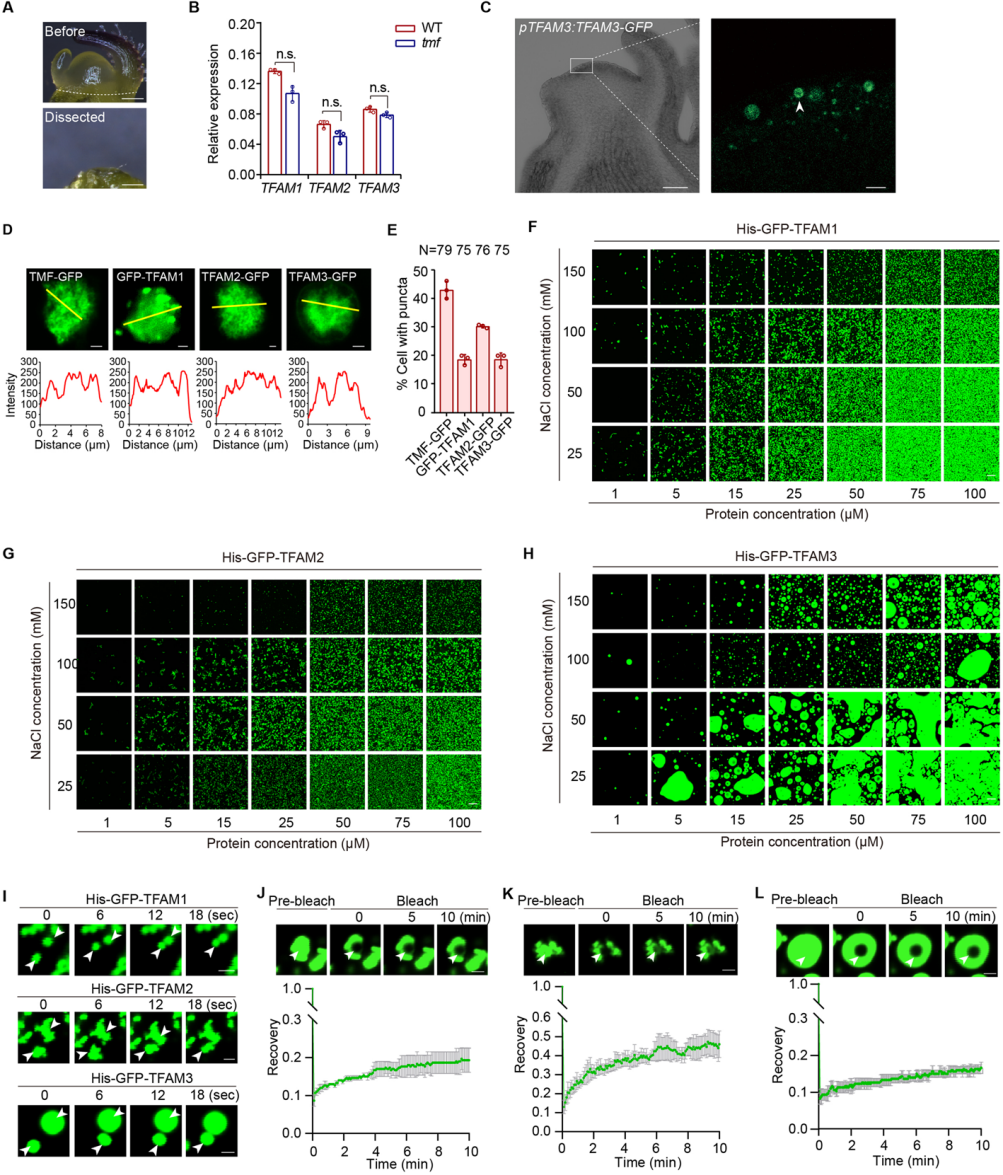
TFAM proteins undergo phase separation in vivo and in vitro. **A** Stereoscope images of the micro-dissected transitional meristem for ddPCR. White dashed line indicates the dissection position. Scale bars, 100 μm. **B** The relative expression of *TFAM1*, *TFAM2* and *TFAM3* were normalized to *UBIQUITIN* (*UBI*), respectively. Data are presented as three technical replicates. Data are means ±SD (*n* = 3, Student *t*-test). Three independent experiments with similar results were carried out. **C** Images showing the TFAM3 condensates in the shoot apical meristem of *pTFAM3:TFAM3-GFP* transgenic plants. Scale bars, 50 μm (left), 5 μm (right). **D** Subcellular localization of TFAMs showing condensates in the nucleus of tomato cells (upper) and fluorescence intensity of indicated yellow lines (bottom). Scale bars, 2 μm. **E** Quantitative data showing the percentage of cells with condensates for GFP fusion proteins of TMF, TFAM1, TFAM2, and TFAM3 in the nucleus. Data are presented as three biological replicates ±SD (*n* = 79, 75, 76, 75). **F**–**H** Phase separation of GFP-TFAM1 (**F**), GFP-TFAM2 (**G**), and GFP-TFAM3 (**H**) under the different combinations of indicated concentrations for NaCl and proteins. Scale bars, 20 μm. Three independent experiments with similar results were performed. **I** Representative images from three independent fusion events showing the liquidity of GFP-TFAM1 (upper), GFP-TFAM2 (middle), and GFP-TFAM3 (bottom) during phase separated droplets formation. Protein concentration, 15 μM; NaCl concentration, 25 mM. Scale bars, 2 μm. **J**–**L** Representative images and quantification data of FRAP analysis for GFP-TFAM1 (**J**), GFP-TFAM2 (**K**), and GFP-TFAM3 (**L**). Data are means of three independent FRAP events. Scale bars, 2 μm

The synergistic interactions and overlapped functions of TMF and TFAMs prompt us to investigate molecular mechanism underlying the genetic canalization. One type of paralogous compensation to achieve genetic canalization is “active compensation,” whereby one or more paralogs are transcriptionally upregulated to compensate the compromised activity of another [30]. To test if that is the case for *TFAM* genes, we examined the transcriptional level of *TFAM1/2/3* in *tmf* mutant plants by quantitative Real-Time PCR (qRT-PCR) and found that loss of *TMF* fails to cause transcriptional upregulation of *TFAM1/2/3* for compensation (Additional file 1: Fig. S2). To confirm this result and detect the transcripts at single cell resolution, we micro-dissected the fresh shoot apical meristems and performed droplet digital PCR (ddPCR) assay (Methods), which showed that loss of *TMF* did not cause up-regulation of the *TFAM* genes (Fig. 4B), ruling out the likelihood of transcriptional compensation.

We then investigate behaviors of the TFAM proteins. The fact that TMF undergoes phase separation and forms condensates in the nucleus in tomato cells prompted us to generate transgenic lines carrying TFAM3-GFP fusion protein driven by TFAM3 native promoter. We observed TFAM3-GFP puncta in the shoot apical meristem (Fig. 4C), suggesting the formation of phase-separated condensates. We then took advantage of our previously established tomato protoplast cell system to express and visualize TFAM-GFP fusion proteins [24]. Confocal imaging showed that all three TFAM proteins exclusively localized in the nucleus as TMF did (Fig. 4D). Interestingly, the GFP signals in the nucleus show high heterogeneity characteristic of an aggregate or condensate state, resembling the punctate localization pattern of TMF (Fig. 4E) [24]. The consistency of localization pattern of both TFAM3 and TMF in tomato shoot apical meristem and protoplast cells prompted us to adopt the protoplast system for further investigation to facilitate the observation and comparison of condensation property of TFAM proteins at single cell resolution. Given the fact that TMF undergoes liquid-liquid phase separation to form transcriptional condensates in the nucleus [24], the heterogenous condensation of TFAM proteins in the nucleus is also likely due to protein phase separation.

### TFAM proteins undergo phase separation in vitro

Further analysis of TFAM proteins revealed that, like TMF, all three TFAMs have prion-like intrinsically disorder regions (IDRs), a signature of proteins capable of phase separation (Additional file 1: Fig. S3A). We then recombinantly expressed and purified the GFP-TFAM fusion proteins from *E. coli* (Additional file 1: Fig. S3B). We used the purified proteins to perform an in vitro phase separation assay and generated a phase diagram by systematically changing protein and salt concentrations to assess the conditions that promote condensate formation [24]. Interestingly, while all three TFAM proteins undergo phase separation, they showed variations in such properties. Like TMF, TFAM3 readily phase-separated into droplets with a relatively regular spherical shape. However, TFAM1 and TFAM2 formed more irregular filamentous assemblies. Both the filaments and droplets are stable during the period of observation (Fig. 4F-H; Additional file 1: Fig. S3C). The phase diagram showed a progressive increase of density and size of the condensates formed by phase-separated TFAMs as the protein concentration increases and the salt concentration keeps constant (Fig. 4F–H). In contrast, the condensate abundance decreased with the increase of salt concentration when the protein concentration is constant, indicating the phase separation behavior is sensitive to both salt and protein concentrations (Fig. 4F–H). In particular, TFAM3 proteins started to form visible spherical droplets at a concentration of 1 μM in a buffer with 150 mM NaCl (a physiologically relevant salt concentration), and the droplets rapidly fused together to form large droplet clusters as protein concentration increases (Fig. 4H). The phase separation of TFAM2 seems more sensitive to salt than that of TFAM1 and TFAM3, suggesting their distinct characteristics. Deletion of IDRs of three TFAMs dramatically decreased droplets formation, indicating that IDRs are essential for phase separation of TFAM1, 2 and 3 (Additional file 1: Fig. S3D).

We then captured the fusion process of the condensates using time-lapse microscopy. The results show that the condensates formed by all three TFAM proteins can rapidly fuse by necking and relaxation to form a larger one upon intersection of two droplets (Fig. 4I; Additional file 2: Movies 1; Additional file 3: Movie 2; Additional file 4: Movie 3), suggesting their dynamic compatibility. To validate this, we performed fluorescence recovery after photobleaching (FRAP) analysis to bleach the centers of large droplets and monitored recovery. The bleached pots started to recover after several seconds, and eventually reached around 20% to 50% recovery of the originally detected signal intensity after several minutes (Fig. 4 J-L; Additional file 5: Movie 4; Additional file 6: Movie 5; Additional file 7: Movie 6). Together, our findings demonstrated that all three TFAM proteins undergo phase separation in vitro and they show varying phase separation capabilities when existed independently.

### TMF interacts with TFAMs to form heterotypic condensates

**Fig. 5:**
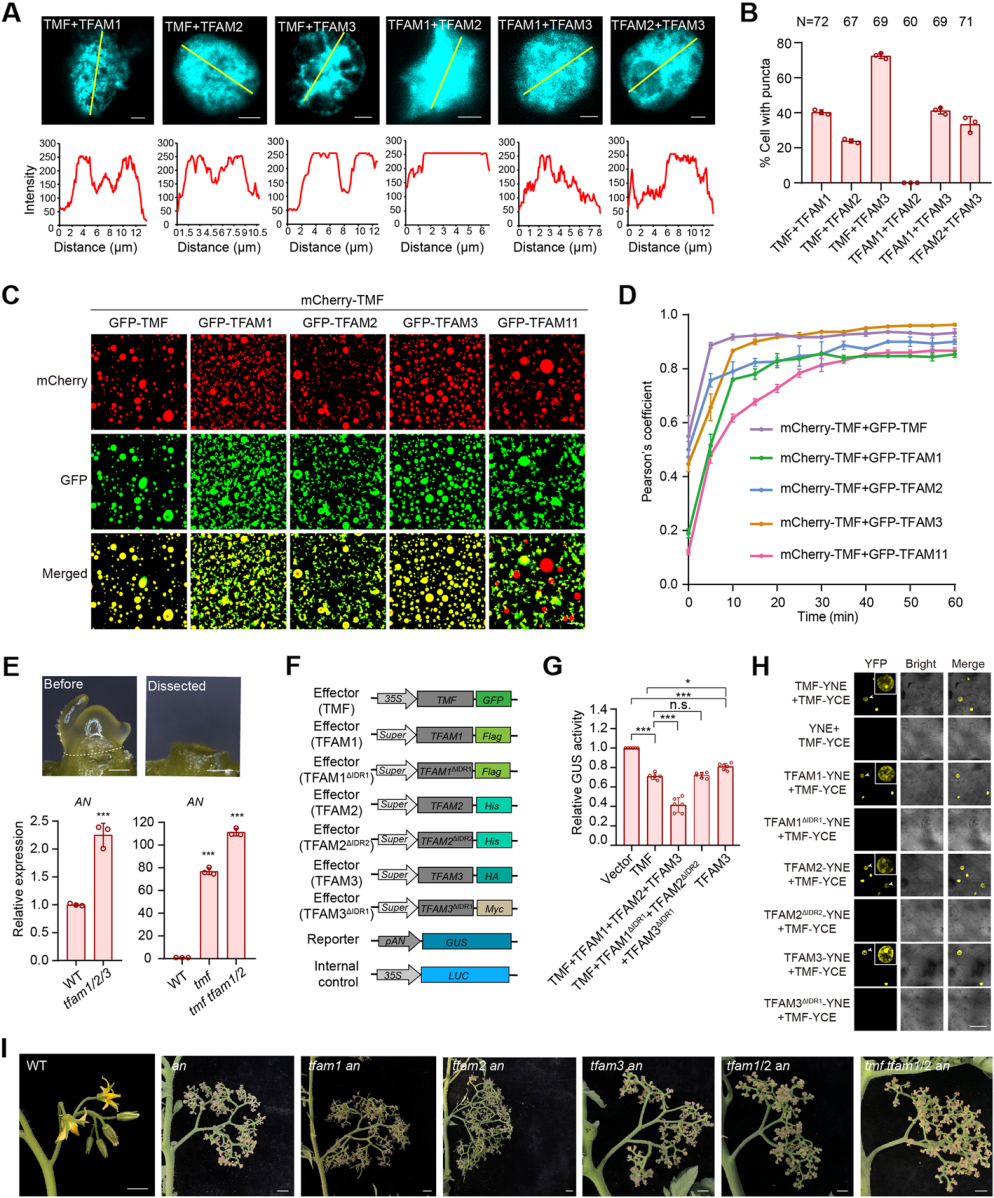
Transcriptional condensates formed by heterotypic interaction between paralogous TFAM proteins repress *AN* expression to synchronize flowering. **A** Representative images (upper) showing the interactions between ALOG proteins in BiFC assays. The fluorescence intensity of indicated yellow lines (bottom) showing the heterogenous condensates formed from interactions between TFAM proteins. Scale bars, 2 μm. **B** Quantitative data showing the percentage of cells with condensates formed by interactions between TFAM proteins in nuclei. Data are presented as three biological replicates ± SD (*n* = 72, 67, 69, 60, 69, 71). **C** Cross-mixing phase separation reactions using recombinantly expressed mCherry-TMF fusion proteins and GFP fusion proteins of TFAMs. Protein concentration, 15 μM; NaCl concentration, 25 mM. Scale bar, 20 μm. **D** Quantification data showing the intersection between TMF droplets and TFAM1/2/3/11 droplets, respectively. Pearson’s coefficient was calculated by ImageJ. Data are presented as three biological replicates ±SD (*n* = 3). **E** Stereoscope images (upper) of the micro-dissected transitional meristem for real-time PCR (bottom). White dashed line indicates the dissection position. The relative expression of *AN* was normalized to WT using *UBIQUITIN* (*UBI*) as an internal control. Data are presented as three replicates ± SD (*n* = 3, ***P < 0.001, Student *t*-test). Three independent experiments with similar results were carried out. Scale bars, 100 μm. **F** Schematics of constructs used to analyze transcriptional activity. **G** Transcriptional repression of *AN* by transcriptional condensates formed by TMF, TFAM and variant TFAM proteins as indicated. The ratio of GUS to LUC indicates relative transcriptional activity. LUC served as an internal control. Data are presented as six biological replicates from two independent experiments. Data are means ±SD (*n* = 6, ***P < 0.001, Student *t*-test). **H** BiFC assays visualizing the condensates formed by TMF and TFAMs in tobacco leaves. Scale bar, 50 μm. **I** Representative images for primary inflorescences of WT, *an* and higher-order mutants of *an* and *tfams*. Scale bars, 1 cm

Given that the four ALOG proteins share phase separation property, we then explore if TMF interacts with three TFAMs to form a protein complex that enables the formation of heterotypic condensates. We took advantage of the bimolecular fluorescence complementation (BiFC) assay, by which we can simultaneously detect protein-protein interactions and analyze phase-separated condensates in living tomato cells. We performed the pairwise interaction tests between TMF and three TFAMs. The results showed that four proteins interacted with each other in the nucleus, supporting the notion of heterotypic protein complexes. Image analysis of heterogeneity of the fluorescence intensity and quantification of the cells with condensates indicated that almost all combinations of interacting pairs induce formation of biological condensates, except for the combination of TFAM1 and TFAM2, whose interaction displays homogenous fluorescence signals (Fig. 5A, B; Additional file 1: Fig. S4).

To validate the interactions in vitro and monitor the phase separation behavior during interactions without disturbance from other potential interacting partners, we recombinantly expressed and purified mCherry-TMF (red fluorescence) and GFP-TFAM (green fluorescence) proteins to perform cross-mixing phase separation reactions [31]. Note that while fusion of distinct fluorescence proteins represents a reliable and the most widely used approach to test protein-protein co-localization or interactions, labeling proteins with fluorescent dyes also serves as an effective way to perform such assays, especially when fusion of fluorescence protein might interfere function of target proteins. The fact that stable transgenic expression of TMF/TFAM tagged with fluorescent protein can fully complement the phenotypes of mutant plants demonstrated that fusion of fluorescent protein did not interfere functions of TMF/TFAM. We therefore used fusion of fluorescent protein to facilitate real-time visualization of droplet interactions. Apparently, TMF can coexist with itself to form the perfectly homotypic droplets (Fig. 5C). However, it largely but not fully merges with TFAM1 and TFAM2 droplets (Fig. 5C). Surprisingly, TMF shows the most remarkable compatibility with TFAM3 in the same droplets, almost identical to the degree of TMF with itself (Fig. 5C), suggesting their tight interactions and cognate property of protein multivalence that promote formation of heterotypic droplets. In contrast, TMF rarely merged with TFAM11 to form heterotypic droplets at the same incubation time as TFAM1, 2 or 3. Instead, TMF-TFAM11 mixtures preferred to exist as a two-phase regime (Fig. 5C). To further investigate the co-existence and fusion properties of TMF-TFAMs droplets for formation of heterotypic condensates, we performed time-lapse imaging to monitor various combinations of TMF-TFAM protein mixtures. Upon mixing, the TMF droplets can quickly intersect with the TFAM3 droplets to fuse together within 15 min, showing the fastest heterotypic condensation among all the TMF-TFAM mixtures. While the TMF-TFAM1 and TMF-TFAM2 mixtures showed slower fusions, they can also largely merge (Fig. 5D; Additional file 1: Fig. S5). Interestingly, TMF barely fuse with TFAM11 at the same incubation time as TFAM3, only showing partial fusion after a much longer incubation time (Fig. 5D; Additional file 1: Fig. S5). This inefficient compatibility of TFAM11 protein with TMF and other TFAMs is consistent with its dispensable role in regulating flowering and inflorescence architecture, providing a molecular support from a protein behavior perspective. As the middle ALOG domain is highly conserved among all TMF family proteins (Additional file 1: Fig. S6), the difference of fusion and co-existence property between TMF and TFAM1, 2, 3 and 11 might be due to the variance of amino acid composition of their IDR regions.

### The ALOG transcriptional condensates repress *AN* expression to synchronize flowering

We recently reported that TMF directly targets floral identity gene *AN* to repress its expression in meristems before flowering transition [24]. To test if TMF acts together with TFAM1/2/3 to target *AN*, we micro-dissected transitional meristems from WT, *tfam1/2/3, tmf* and *tmf tfam1/2* plants for qRT-PCR analysis (Fig. 5E upper). The results showed that *AN* expression was precociously activated in *tfam1/2/3* compared to WT (Fig. 5E bottom). As previously reported and shown here, *AN* prematurely and dramatically upregulated in *tmf*. This effect is significantly enhanced in *tmf tfam1/2* triple mutant (Fig. 5E bottom), indicating that TMF and TFAM1/2/3 synergistically repress *AN* expression in SAMs before floral transition. To validate if the transcriptional repression is a direct action, we performed a series of transcriptional activity assays using beta-glucuronidase (GUS)–luciferase (LUC) dual reporter system in tobacco leaves, where promoter sequence of *AN* was fused with GUS to serve as a reporter, and various combinations of co-expressed TMF and TFAMs served as effectors [24] (Fig. 5F). The assays showed that co-expression of three TFAMs significantly reinforced TMF’s repression of *AN* transcription (Fig. 5G; Additional file 1: Fig. S7A). We then took advantage of the same tobacco living plant system to test if this enhancement of transcriptional repression relies on the formation of heterotypic transcriptional condensates. The BiFC assay indicated that TMF indeed interacted with TFAMs to form transcriptional condensates in the nucleus (Fig. 5H). Notably, the Co-immunoprecipitation assay showed that TMF interacted with three TFAMs to form a heterotypic protein complex (Additional file 1: Fig. S7C). However, deletion of IDRs of TFAMs not only abolished the heterotypic interaction and formation of biomolecular condensates (Fig. 5H; Additional file 1: Fig. S7C) but also eliminated the enhanced transcriptional repression on *AN* (Fig. 5G), demonstrating the essentiality of formation of TMF-TFAM heterotypic condensates in robust control of transcriptional repression on *AN*.

The aforementioned molecular evidences were then confirmed by extensive genetic analysis. As *anantha* (*an*) homozygous mutant repeatedly over-proliferates axillary inflorescence meristems but never forms normal flowers [13], we crossed *an* heterozygous mutant with single and high-order mutants of *tfam* and *tmf*. By screening progeny from F_2_ plants, we obtained *tfam1 an*, *tfam2 an*, *tfam3 an*, *tfam1/2 an*, and *tmf tfam1/2 an* mutants. Inflorescences of these mutants were indistinguishable from *an* mutant, suggesting that *an* is completely epistatic to *tmf* and *tfam* mutants (Fig. 5I). Together, these results demonstrate that transcriptional condensates formed by four paralogous proteins of ALOG family precisely control meristem maturation by directly repressing *AN* expression, which ensures adequate vegetative growth before floral transition and compound inflorescences production.

### Transcriptional repression capacity of TFAMs on *AN* relies on IDR variation

Identification of *AN* as a target gene of TFAM condensates provides a molecular reporter for transcriptional activity evaluation. Together with phase separation capacity comparison using recombinantly expressed proteins, we can explore the molecular mechanism underlying the differences of TFAM condensates and the resulting phenotypes. Although the expression level of *TFAM* genes are different in the SAM (Fig. 1A), it cannot exclusively explain the phenotypic severity differences of their mutants since loss of *TFAM11* did not show stronger phenotypes than other three *tfam* mutants despite its transcription abundance is comparable to or even higher than other three TFAMs (Fig. 1A). To validate this, we used *AN* as a reporter gene to evaluate the differences of the transcriptional repression capacity via GUS–LUC dual reporter assay in living plants. *tfam3* shows the closest phenotypes to *tmf* among all *tfam* mutants, we therefore used 35S constitutive promoter to increase *TFAM3*’s expression to the equal level of *TMF*, but it still fails to reach the comparable transcriptional repression effect on *AN* (Fig. 5G; Additional file 1: Fig. S7B). These genetic and molecular evidence suggested that the variation in protein behavior contributes to the genetic robustness achieved by paralogous interaction.

**Fig. 6:**
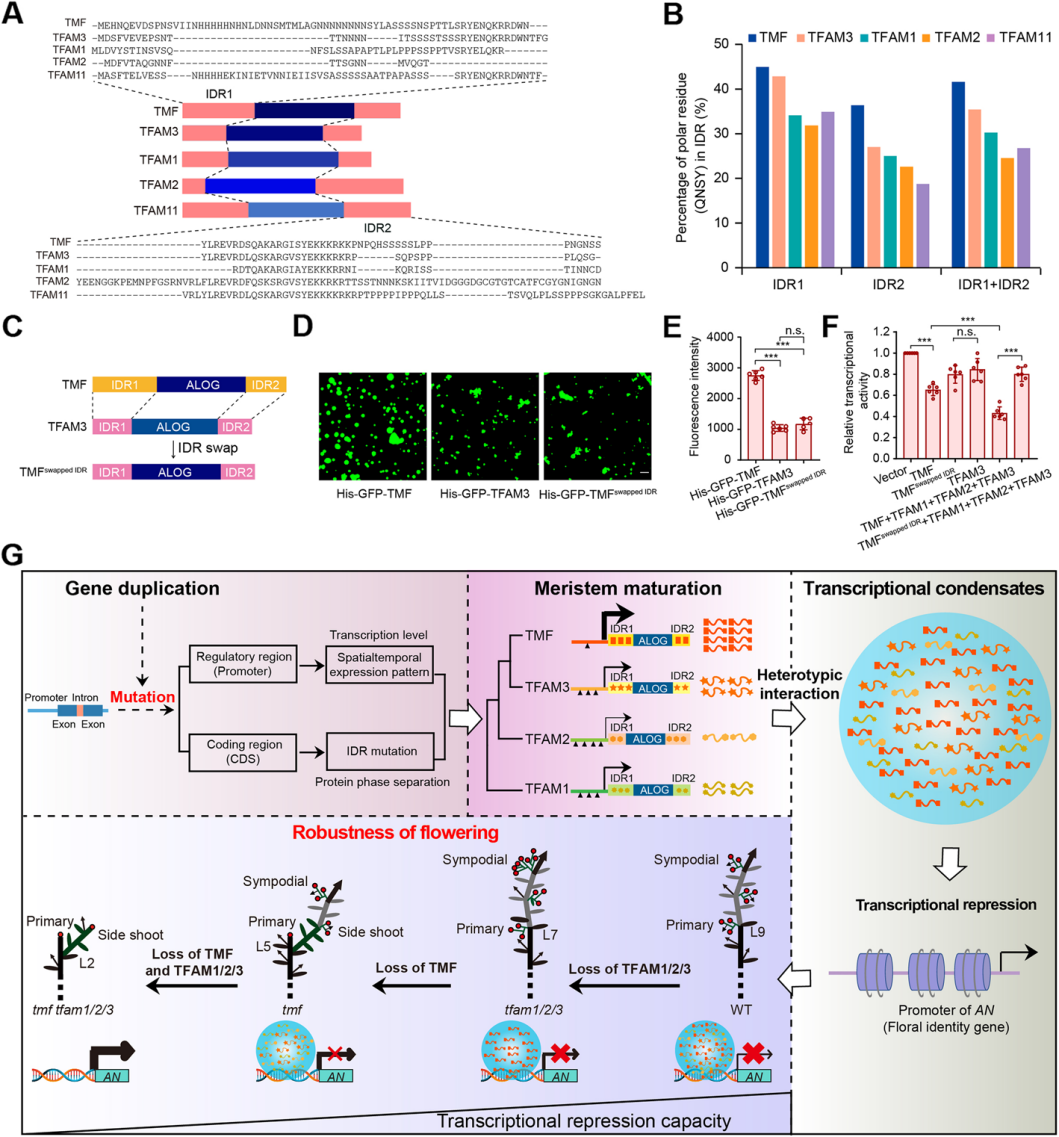
IDR variation determines phase separation and transcription regulating activity of TMF family proteins. **A** IDR comparisons between TMF family proteins. **B** Quantification of polar residues for Gln (Q), Asn (N), Ser (S), and Tyr (Y) in IDRs. **C** Schematic of IDR swap between TMF and TFAM3. **D**, **E** Representative images (**D**) and quantification (**E**) of phase separation for TMF, TFAM3 and IDR swapped TMF proteins in vitro. Protein concentration, 5 μM. NaCl concentration, 50 mM. Scale bar, 20 μm. Data are presented as six biological replicates. Data are means ± SD (*n* = 6, ***P < 0.001, Student *t*-test). **F** Transcriptional repression of *AN* by transcriptional condensates formed from TMF, TMF^swapped IDR^, and TFAM proteins. The ratio of GUS to LUC indicates relative transcriptional activity. LUC served as an internal control. Data are presented as six biological replicates from two independent experiments. Data are means ±SD (*n* = 6, ***P < 0.001, Student *t*-test). **G** Working model for flowering robustness achieved by heterotypic interaction and transcriptional condensation of ALOG family paralogs

Given the prion-like IDR regions are common driving force for protein phase separation, we first analyzed the IDRs of TFAMs and found that, comparing to the ALOG domains, they varied considerably both in length and amino acid composition (Fig. 6A). Enrichment of polar amino acids, such as glutamine, asparagine, serine, and tyrosine, and their low-complexity arrangements mark the phase separation potential of a protein [32–34]. Consistently, the TFAM protein with the highest abundance of these polar amino acids shows the strongest phase separation (Fig. 6B and Fig. 4). In particular, TMF and TFAM3 have asparagine-repeats and serine-repeats in their IDR regions, respectively (Fig. 6A), consistent with their higher phase separation capacities. The fact that IDR composition and phase separation capacity mirrored transcriptional repression ability of TFAMs, for example, the strongest phase separation of TMF having the greatest transcriptional repression effect on *AN* (Fig. 5G; Additional file 1: Fig. S7A), suggested that the IDR variation might account for the differences in transcriptional repression abilities among TFAMs. To confirm this, we precisely swapped the IDR regions between TMF and TFAM3 and found that the chimeric TMF^swapped IDR^ lost the phase separation capacity of normal TMF, but resembled phase separation behavior of TFAM3 (Fig. 6C-E). The transcriptional activity of IDR-swapped proteins were then investigated in living plants using GUS–LUC dual reporter assays. As expected, TMF^swapped IDR^ failed to repress *AN* expression as TMF did, instead, it mimicked TFAM3 (Fig. 6F). Importantly, when co-expression with all other three TFAMs, it never reached the dominant role of TMF in the heterotypic transcriptional condensates on repressing *AN* (Fig. 6F), demonstrating that the IDR variation causes differences in phase separation capacity and thus in transcriptional activity.

## Discussion

Flowering plants are the youngest and the most diverse land plants. Their genomes have experienced extensive genome-wide and regional duplications. The evolutionary fates of duplicated genes shape phenotypic stability and diversity, such as inducing disease resistance and adaptation to stress [35, 36]. By default, paralogs are functional redundant immediately following duplication, and their interactions set up the stage for genetic canalization [37]. Here, we uncover a new canalization mechanism driven by transcriptional condensation via heterotypic interaction and phase separation of plant ALOG family paralogous proteins, which enables robust control of SAM maturation for flowering transition and compound inflorescence production in tomato.

Our findings support a model as shown in Fig. 5G. Gene duplication expands the ancient ALOG gene family and produce multiple redundant paralogs including TMF and TFAMs. Mutations occurred in *cis*-regulatory and coding regions of IDRs following gene duplications cause different transcription levels and protein behavior among the paralogs (Fig. 6A; Additional file 1: Fig. S8). Specifically, TMF is endowed with the highest transcription level and strongest phase separation, serving as the dominant player to interact with its paralogous partners TFAM1, 2, and 3. The four proteins interact and form heterotypic phase-separated condensates, and the resulting transcriptional condensates bind to the promoter of floral identity gene *AN* to suppress it expression during vegetative meristem stages. This phase-separation based repressing program by a collection of closely related gene family members “canalizes” the vegetative state maintenance system to ensure proper proliferation of enough stem cells before the crucial transition to flowering and reproductive success. This well-established canalization process by multiple participants not only ensures the robustness of the system but also releases selective pressure on individual genes, creating flexibility for sub-functionalization or neofunctionalization of the players, as suggested by potential specialized functions of certain TFAMs in other developmental processes. For example, TFAM1 acquires new function in floral organ morphogenesis and abscission [10] (Additional file 1: Fig. S9). Our finding that both transcription level and protein behavior contribute to the functional significance of paralogs within a gene family reminds that overemphasis of transcription level might leads to ignore or mischaracterize functions of a gene in specific development processes, particularly when the transcriptome analysis becomes more straightforward and less expensive.

Despite being sessile, plants have successfully propagated and robustly survived in diverse ecosystems. One key to this evolutionary success is their potent capacity to leverage genome duplications for adaption and innovation. Biological robustness, usually achieved by presence of fully or partially redundant parts that result from gene duplication, shields plants from drastic environment perturbations and fuels functional innovation through relaxed negative natural selection [2, 38]. Functional overlap between paralogs allows them to compensate each other's loss, as commonly revealed by aggravating genetic interactions [2]. Through powerful reverse genetic tools like CRISPR/Cas9 mutagenesis, it becomes possible to create a full series of mutant combinations of all ALOG paralogs involved in SAM maturation process to comprehensively dissect the functional overlaps underlying the robustness of this system. For example, losing one or two TMF paralogs, tomato plants can still produce multiple-flowered inflorescences with a subtle early-flowering phenotype (Figs. 1A–C, 6G). However, losing four functional overlapped ALOG genes simultaneously, tomato plants flower extremely early after producing only two true leaves and develop a single-flowered inflorescence with severe floral organ defects, leading to failure in setting fruits and seeds for propagation (Figs. 1D–E, 2A–E, 6G). Our study at genetic and molecular levels demonstrated that a unique heterotypic transcriptional condensation mechanism underpins the coordination of functional overlapped paralogs to achieve this biological robustness.

Approximately 40% of all proteins in eukaryotic organisms are either entirely disordered or contain sizeable regions that are disordered [39, 40]. In this study, variations in IDRs among paralogous proteins are likely due to accumulation of spontaneous mutations under relaxed purifying selection following gene duplication [40], which resulted in variable phase separation capabilities. This in turn leads to varied cognate recognition for complete functional compensation among family members, but also opens the door for functional innovation while maintaining the core function of repressing premature floral transition. IDR-driven protein phase separation represents a type of dynamic and flexible protein behavior that has been recently reported to implicate in acclimation responses to cellular pH levels, heat, and oxidative stress [41–44]. The condensates formed by genealogically related and functional redundant paralogous proteins might have evolved in multicellular organisms to create various “canalized” systems in a much broader context than flowering control. These systems help release purifying selection pressure on individual gene family members and retain cryptic genetic variations as raw materials for genetic, developmental and phenotypic innovations.

## Conclusions

Following gene duplication, paralog expansion and diversification enable phenotypic robustness. Our findings reveal how heterotypic transcriptional condensates formed by paralogous protein interactions and phase separation canalize plant flowering transition. This study uncovers the molecular link between gene duplication caused variation of prion-like intrinsically disordered regions (IDRs) of paralogous proteins and robust transcriptional control of stem cell fate transition.

## Methods

### Plant materials and growth conditions

The *tmf*, *an*, *tfam1*, *tfam2* and *tfam3* and *tfam11* single mutants used in this study are in tomato (*Solanum lycopersicum*) cultivar M82 background. The higher-order mutants for *tfam1/2*, *tfam1/3*, *tfam2/3*, *tfam1/2/3*, *tmf tfam1*, *tmf tfam2*, *tmf tfam3*, *tmf tfam1/2*, *tmf tfam1/3*, and *tmf tfam1/2/3* were produced by crossing using single mutants. The homozygotes were genotyped by digestion of PCR production amplified. Seedlings were grown in growth room at 26 °C, with 45–60% relative humidity under LED (Philips Lighting IBRS) light. Greenhouse plants were grown under natural light supplemented with LED. 16 h light/8 h dark photoperiod was used for seedlings and greenhouse plants.

### Transcriptional activity assay

The GUS–LUC dual reporter system as previous described was used to perform transcription activity assays in vivo [24]. The ALOG proteins fused different tags served as effectors. *pAN:GUS* served as a reporter, and *35S:LUC* served as an internal control as described previously. Co-infiltrated the plasmids of effector and reporter into *N. benthamiana* leaves, and harvested leaves after 60 h. Total proteins were extracted for measuring the activity of GUS and luciferase (LUC) activity using 4-methylumbelliferyl glucuronide (Sigma) and luciferin (Promega) as substrates, respectively. The transcriptional activity was determined by the ratio of GUS/LUC.

### Recombinant protein expression and purification

To generate the constructs for recombinant protein expression, coding sequences of fusion DNA fragments for *GFP-TFAMs*, *GFP-TFAMs*^*△IDR*^, and *mCherry-TMF* were cloned into the vector pQE-80 L. The plasmids were transformed into *E. coli* Rosetta (DE3) competent cells, and positive bacteria cultured in LB were induced by 0.5 mM isopropyl β-D-1-thiogalactopyranoside (IPTG) for 16 h at 16 °C. Collected cells and performed purification using Ni-NTA (GE healthcare) affinity beads as previous described. Buffer exchange and concentration for eluted proteins were performed using ultrafiltration tubes (Vivaspin turbo). Purified proteins were stored in storage buffer (50 mM Tris-HCl, 200 mM NaCl, pH 7.4) at − 80 °C after quick-freezing in liquid nitrogen.

### Phase separation assay and FRAP in vitro

The phase separation assays were performed by dilution of purified proteins into buffer containing 50 mM Tris-HCl (pH 7.4) and various concentrations for NaCl to indicated final concentrations in the figure legends. Purified proteins were centrifuged 10 min at 14,000*g* and transferred supernatants into new tubes to exclude the effects caused by precipitated proteins. To generate phase diagram, diluted phase-separated protein solution was incubated for 15 min at room temperature in a 384-well plate. To perform droplet interaction assay for TMF and TFAMs in vitro, purified proteins dissolved in buffer containing 50 mM Tris-HCl (pH 7.4), 25 mM NaCl as indicated in the figure legends were thoroughly mixed and incubated as indicated time at room temperature in a 384-well plate. Images for droplets and filaments were taken using confocal microscopy (Nikon A1R+) equipped with× 20, × 40, and × 100 oil objectives. Fluorescence was excited at 488 and detected at 500–540 for GFP, excited at 543 nm and detected at 595–635 nm for mCherry.

### Subcellular localization and BiFC assays in tomato protoplasts

To investigate the subcellular localization of TFAM proteins, we generated the constructs. The coding sequences of *TFAM1* and *TFAM3* were amplified and separately cloned into transient expression vector to generate *35S:GFP-TFAM1*, *35S:TFAM3-GFP*, and cloned into pSCYNE (SCN) and pSCYCE (SCC) to generate TFAM3-N-CFP (TFAM3-SCN) and TFAM3-C-CFP (TFAM3-SCC) for BiFC assay. Plasmids for *35S:TMF-GFP*, *35S:TFAM2-GFP*, *TMF-SCC*, *TMF-SCN*, *TFAM1-SCC*, *TFAM1-SCN*, *TFAM2-SCC*, and *TFAM2-SCN* were described as previously. [45] Plasmids were transfected into protoplasts isolated from tomato cotyledons as previous described. Fluorescent signals detection was performed using confocal microscopy (Leica SP5) with × 20, × 40 objectives.

### Co-immunoprecipitation and immunoblotting assays

The Co-IP assays were carried out as previously described [46]. The sequence of GFP-TMF was amplified and cloned into pRI101, and TFAM1/TFAM1^ΔIDR1^-Flag, TFAM2/TFAM2^ΔIDR2^-His, TFAM3-HA, TFAM3^ΔIDR1^-Myc were cloned into pSuper1300, respectively. These constructs were co-transfected into tobacco leaves as indicated. The total proteins were extracted from tobacco leaves with lysis buffer (10 mM Tris-HCl, 150 mM NaCl, 0.5% NP40) supplemented with 2 mM DTT. The samples of co-immunoprecipitation assay were incubated with GFP-Nanoab-Agarose beads (Lablead). Proteins were detected using immunoblotting with anti-GFP (Easybio), anti-HA (Sigma-Aldrich), anti-Flag (Sigma-Aldrich), anti-His (Easybio), anti-Myc (Sigma-Aldrich), and anti-Actin (Easybio), respectively.

### Droplet digital PCR (ddPCR)

The ddPCR was performed as described in the manufacturer’s instructions with the QX200 droplet digital PCR system (Bio-Rad). The droplets were generated by QX200 autoDG droplet digital PCR system (Bio-Rad), and the PCR amplification was carried out with EvaGreen Supermix (Bio-Rad) using the recommended cycling conditions. The positive and negative droplets were detected using QX200 droplet reader (Bio-Rad) and analyzed using QuantaSoft software. Each positive droplet is assigned a value of 1, each negative droplet is assigned a value of 0 (zero) [47].

## Supplementary Information

**Additional file 1: Figure S1.** Quantification of penetrance for *tmf* single mutant and higher-order mutants of *tmf* and *tfams*. **Figure S2.** The qRT-PCR showing transcriptional level of *TFAM1*, *TFAM2* and*TFAM3* in WT and *tmf* mutant plants. **Figure S3.** Protein purification for phase separation analysis *in vitro*. **Figure S4.** BiFC assays showing the interactions between TMF and TFAMs in nucleus of tomato protoplast. **Figure S5.** Images showing the droplets intersection between TMF and TMF (A), TFAM1 (B), TFAM2 (C), TFAM3 (D), TFAM11 (E). **Figure S6.** Schematics illustrating protein domains for TMF and TFAM proteins. **Figure S7.** TMF interacts with TFAMs to form a transcriptional repression complex. **Figure S8.** Alignment of 2kb upstream promoter regions for *TMF*, *TFAM1*, *TFAM2* and *TFAM3* genes. **Figure S9.** *TFAM1* regulates floral organ development and abscission. **Figure S10.** Uncropped images for Western blot gel.

**Additional file 2.** Movie 1.

**Additional file 3.** Movie 2.

**Additional file 4.** Movie 3.

**Additional file 5.** Movie 4.

**Additional file 6.** Movie 5.

**Additional file 7.** Movie 6.

**Additional file 8.** Review history.

## Data Availability

Data for expression pattern of TFAM genes are mined and collected from previous study [7] and public database of Tomato Expression Atlas (https://tea.solgenomics.net/expression_viewer/input). The related original data of microscopy images have been submitted to the Figshare. DOI: 10.6084/m9.figshare.19307450 [48].
