# Supplementary material for: Heterotypic transcriptional condensates formed by prion-like paralogous proteins canalize flowering transition in tomato
Source: Genome Biol. 2022 Mar 14;23:78. doi: 10.1186/s13059-022-02646-6 (PMC8919559; doi:10.1186/s13059-022-02646-6)
Supplement: Supplementary file 1 — Additional file 1: Figure S1. Quantification of penetrance for tmf single mutant and higher-order mutants of tmf and tfams. Figure S2. The qRT-PCR showing transcriptional level of TFAM1, TFAM2 andTFAM3 in WT and tmf mutant plants. Figure S3. Protein purification for phase separation analysis in vitro. Figure S4. BiFC assays showing the interactions between TMF and TFAMs in nucleus of tomato protoplast. Figure S5. Images showing the droplets intersection between TMF and TMF (A), TFAM1 (B), TFAM2 (C), TFAM3 (D), TFAM11 (E). Figure S6. Schematics illustrating protein domains for TMF and TFAM proteins. Figure S7. TMF interacts with TFAMs to form a transcriptional repression complex. Figure S8. Alignment of 2kb upstream promoter regions for TMF, TFAM1, TFAM2 and TFAM3 genes. Figure S9. TFAM1 regulates floral organ development and abscission. Figure S10. Uncropped images for Western blot gel. [file 13059_2022_2646_MOESM1_ESM.docx]

**Additional file 1 for**

**Heterotypic transcriptional condensates formed by prion-like paralogous proteins canalize flowering transition in tomato**

Xiaozhen Huang^a, c, 1^, Nan Xiao^a, b, c,1^, Yupan Zou ^a, c^ , Yue Xie^a, b, c^, Lingli Tang^a, b, c^, Yueqin Zhang^a, c, d^, Yuan Yu^a, b, c^, Yiting Li^a ,b, c^ and Cao Xu^a, c, 2^

^a^State Key Laboratory of Plant Genomics, National Center for Plant Gene Research (Beijing), Institute of Genetics and Developmental Biology, The Innovative Academy of Seed Design, Chinese Academy of Sciences, Beijing, China; ^b^University of Chinese Academy of Sciences, Beijing, China; ^c^CAS-JIC Centre of Excellence for Plant and Microbial Science (CEPAMS), Institute of Genetics and Developmental Biology, Chinese Academy of Sciences, Beijing, China; ^d^College of Coastal Agricultural Sciences Guangdong Ocean University, Zhanjiang, China.

^1^These authors contributed equally to this work.

^2^ To whom correspondence may be addressed. Email: [caoxu@genetics.ac.cn](mailto:caoxu@genetics.ac.cn).

**This file includes:**

**Figures S1-S10**

**Movies 1-6**

**Movie 1.** Fusion for two phase-separated GFP-TFAM1 filaments.

**Movie 2.** Fusion for two phase-separated GFP-TFAM2 filaments.

**Movie 3.** Fusion for two phase-separated GFP-TFAM3 droplets.

**Movie 4.** FRAP analysis of phase-separated GFP-TFAM1 *in vitro*.

**Movie 5.** FRAP analysis of phase-separated GFP-TFAM2 *in vitro*.

**Movie 6.** FRAP analysis of phase-separated GFP-TFAM3 *in vitro*.


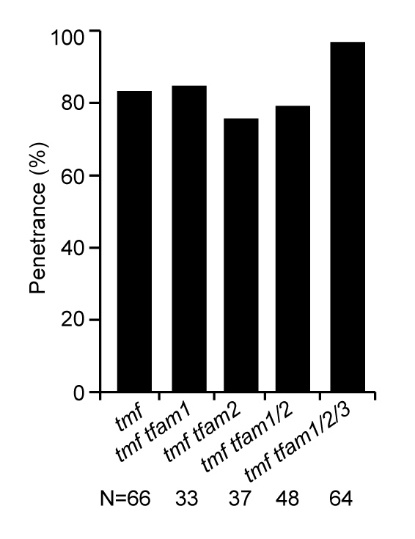


**Figure S1. Quantification of penetrance for *tmf* single mutant and higher-order mutants of *tmf* and *tfams*.**


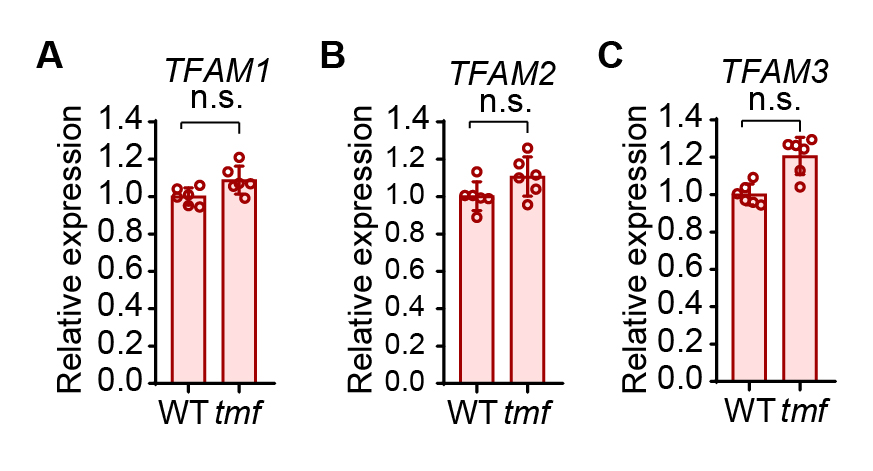


**Figure S2. The qRT-PCR showing transcriptional level of *TFAM1*, *TFAM2* and*TFAM3* in WT and *tmf* mutant plants.** The relative expression of *TFAM1*, *TFAM2* and *TFAM3* were normalized to WT using *UBIQUITIN* (*UBI*) as an internal control, respectively. Data are presented as six replicates from two independent experiments. Data are means ±SD (n=6, Student *t*-test). Three independent experiments with similar results were carried out.


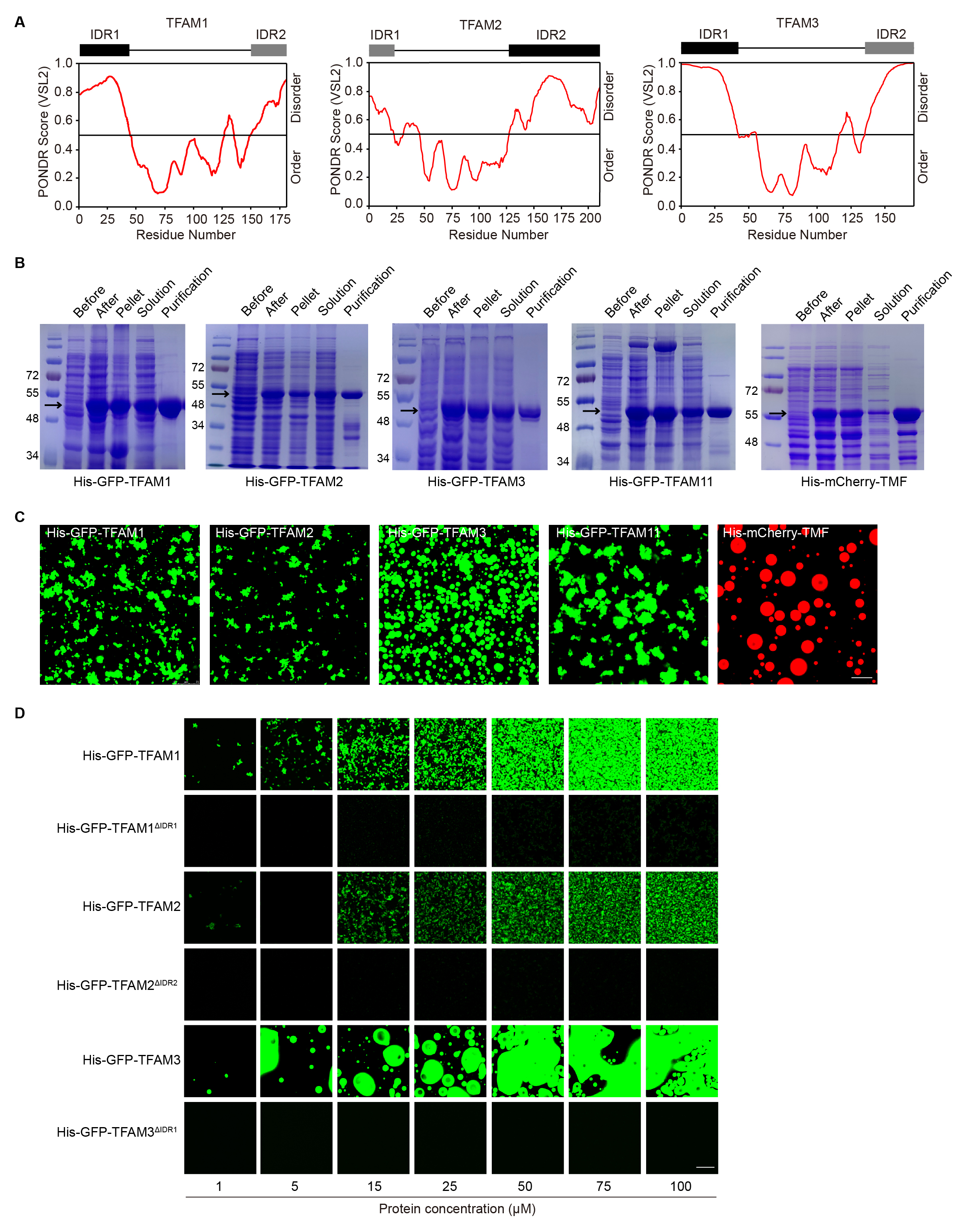


**Figure S3.** **Protein purification for phase separation analysis *in vitro*.** (A) Graphs showing IDRs of TFAM proteins. (B) SDS –PAGE gels showing the induction and purification for His-GFP or His-mCherry fused proteins. The black arrows indicate target bands of proteins, respectively. (C) Images of phase separation for His-GFP-TFAMs and His-mCherry-TMF used in this study. Proteins concentration, 15 μM. NaCl concentration, 25 mM. Scale bar, 20 μm. (D) Representative confocal images showing the effects of IDR deletion on the phase separation for TFAM proteins. NaCl concentration, 25 mM. Scale bar, 20 μm.


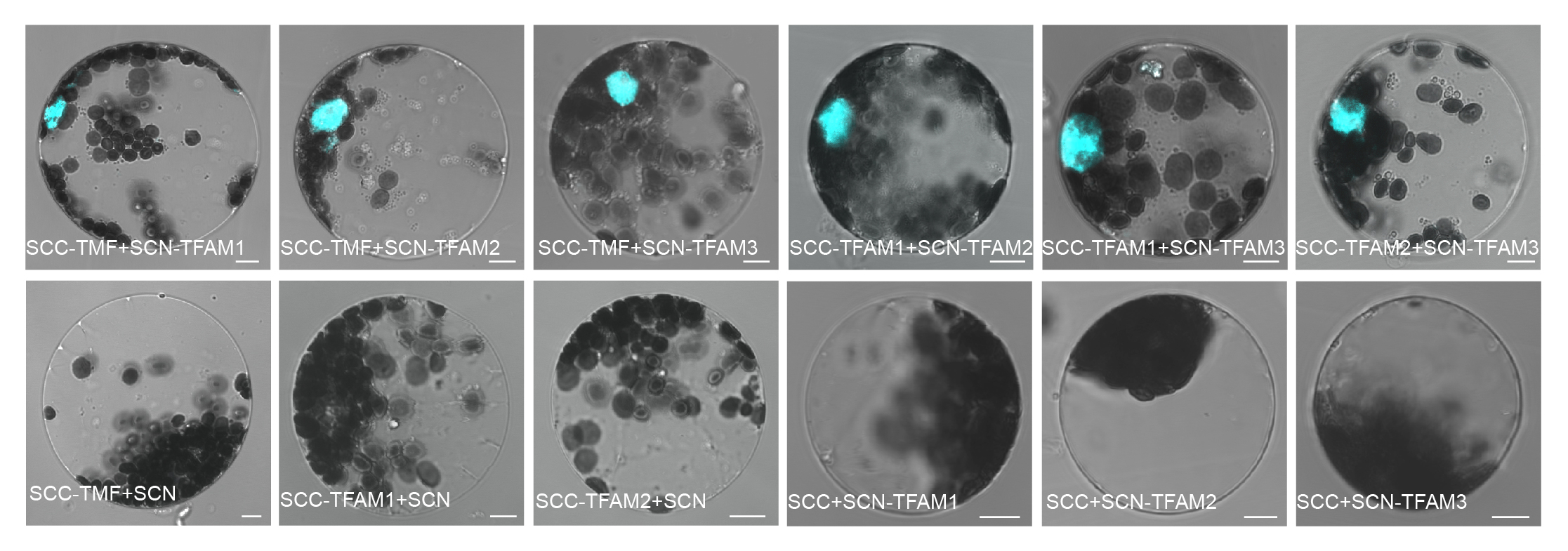


**Figure S4.** **BiFC assays showing the interactions between TMF and TFAMs in nucleus of tomato protoplast**. Scale bars, 7.5 μm.


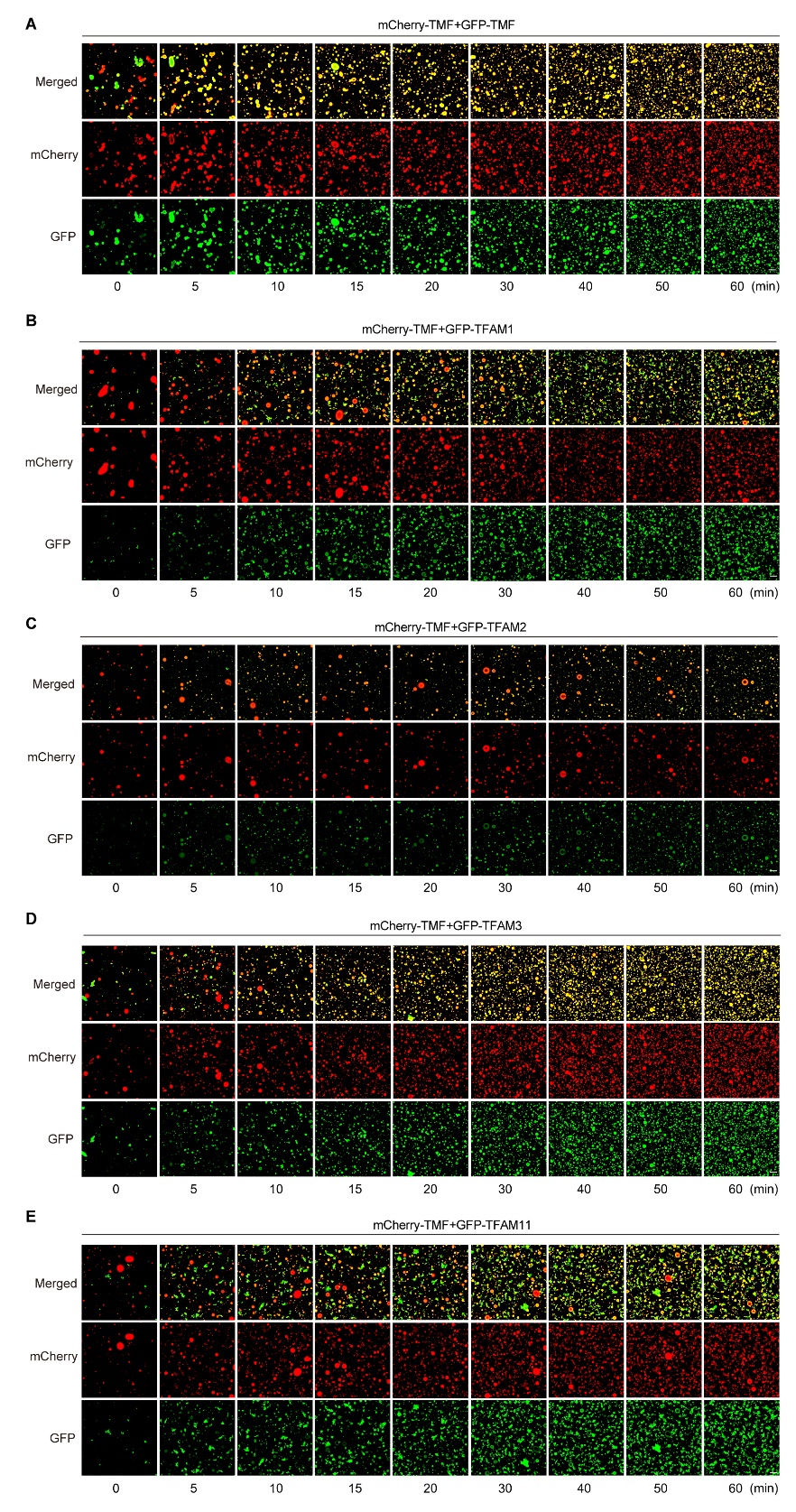


**Figure S5. Images showing the droplets intersection between TMF and TMF (A), TFAM1 (B), TFAM2 (C), TFAM3 (D), TFAM11 (E)**. Proteins concentration, 10 μM. NaCl concentration, 25 mM. Scale bars, 20 μm.


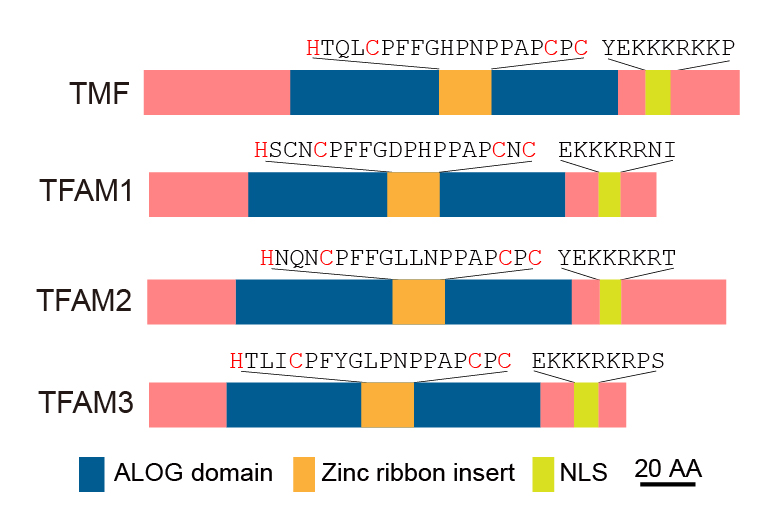


**Figure S6. Schematics illustrating protein domains for TMF and TFAM proteins**. Both of TMF and TFAM proteins include a conserved ALOG domain with a zinc ribbon insert and a nuclear localization signal (NLS).


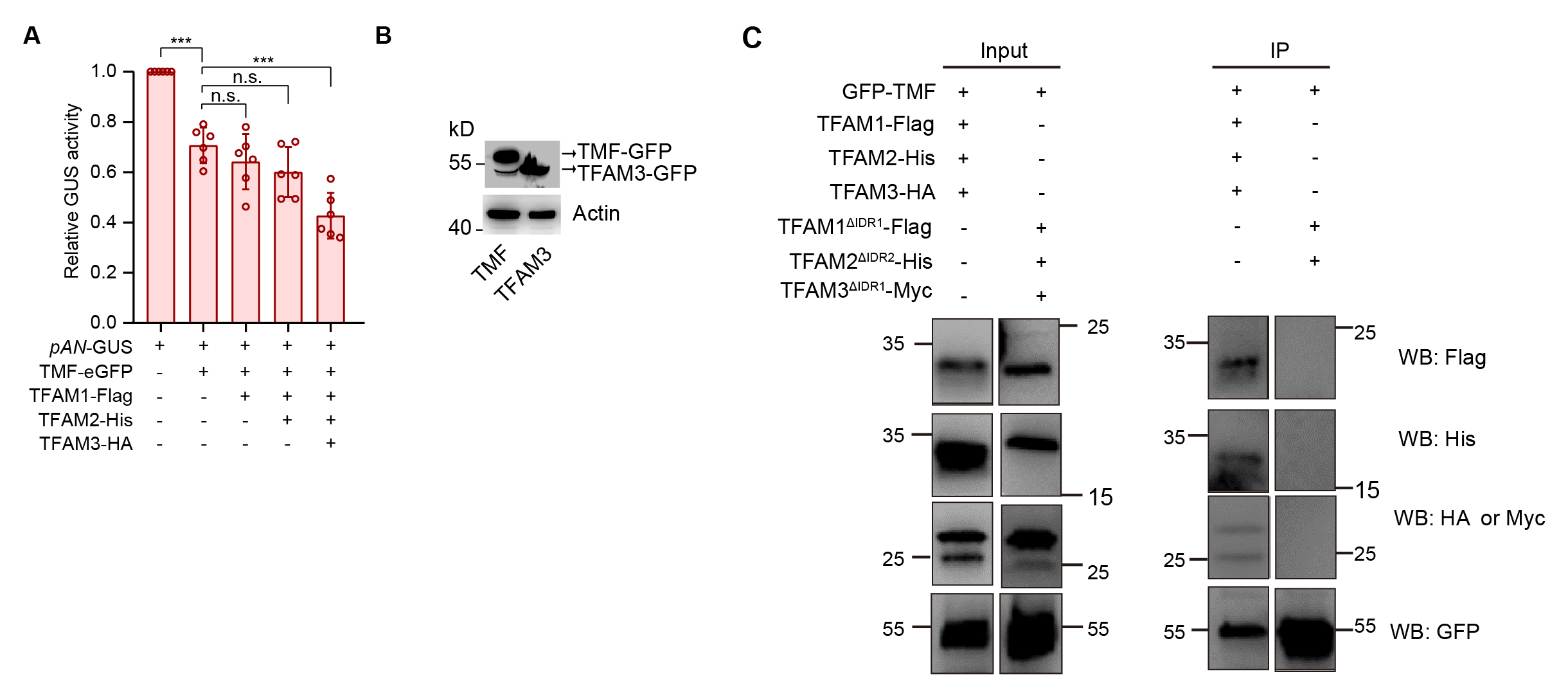


**Figure S7. TMF interacts with TFAMs to form a transcriptional repression complex.** (A) Transcriptional repression of *AN* by transcriptional condensates formed from TMF and TFAM proteins. The ratio of GUS to LUC indicates relative transcriptional activity. LUC served as an internal control. Data are presented as six biological replicates from two independent experiments. Data are means ±SD (n=6, *^***^P*<0.001, Student *t*-test). (B) Immunoblot analysis showing comparable expression level of TMF and TFAM3 proteins for transcriptional activity assays. (C) Co-immunoprecipitation (Co-IP) assays showing that IDR is required for the interaction between ALOG proteins. GFP-TMF as a bait was immunoprecipitated with anti-GFP beads, and TFAMs or variant TFAMs as prey were detected with anti-Flag, anti-His, anti-HA, anti-Myc, respectively.


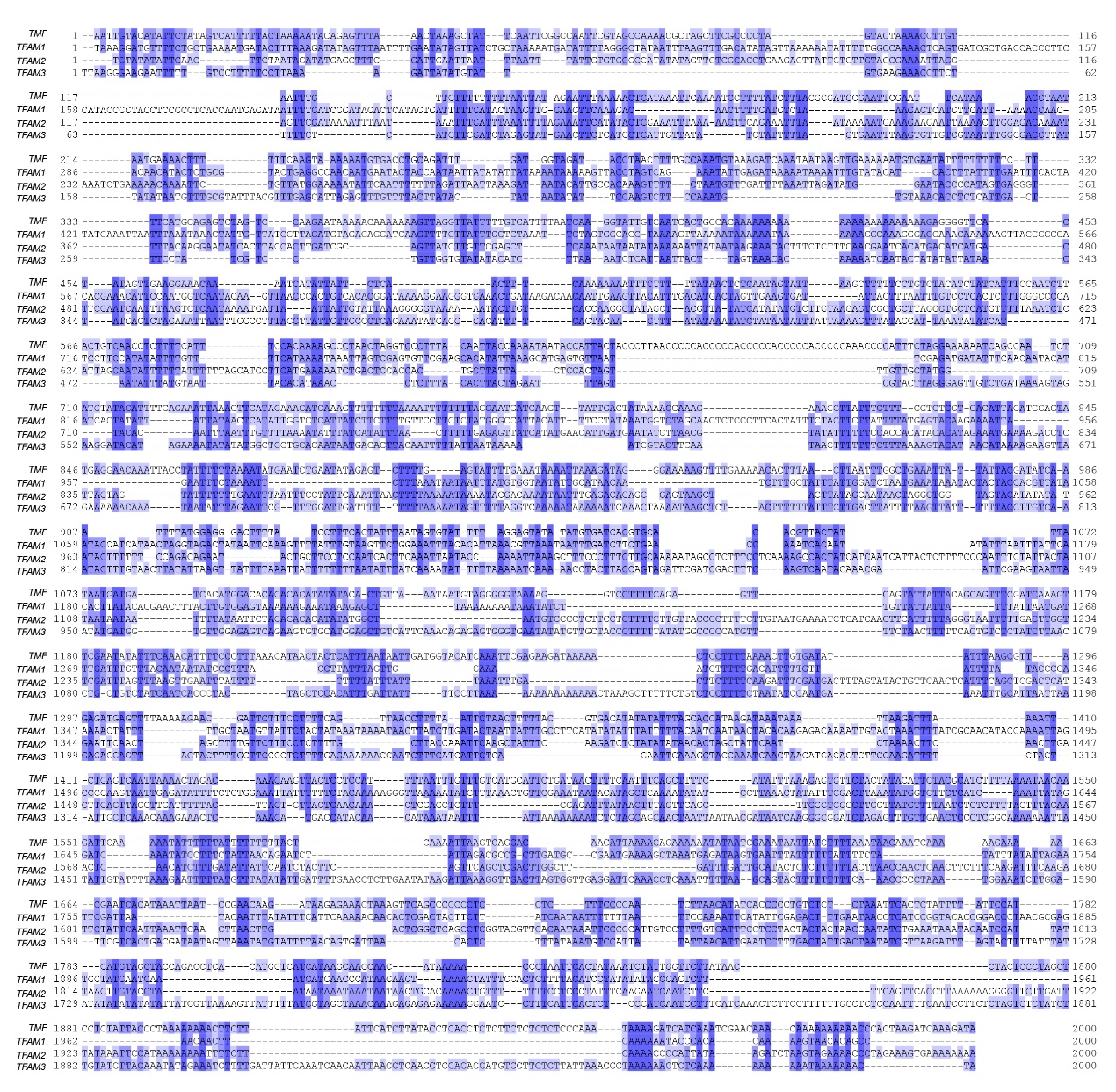


**Figure S8. Alignment of 2kb upstream promoter regions for *TMF*, *TFAM1*, *TFAM2* and *TFAM3* genes**. Different shades of blue showing the percentage identity of bases.


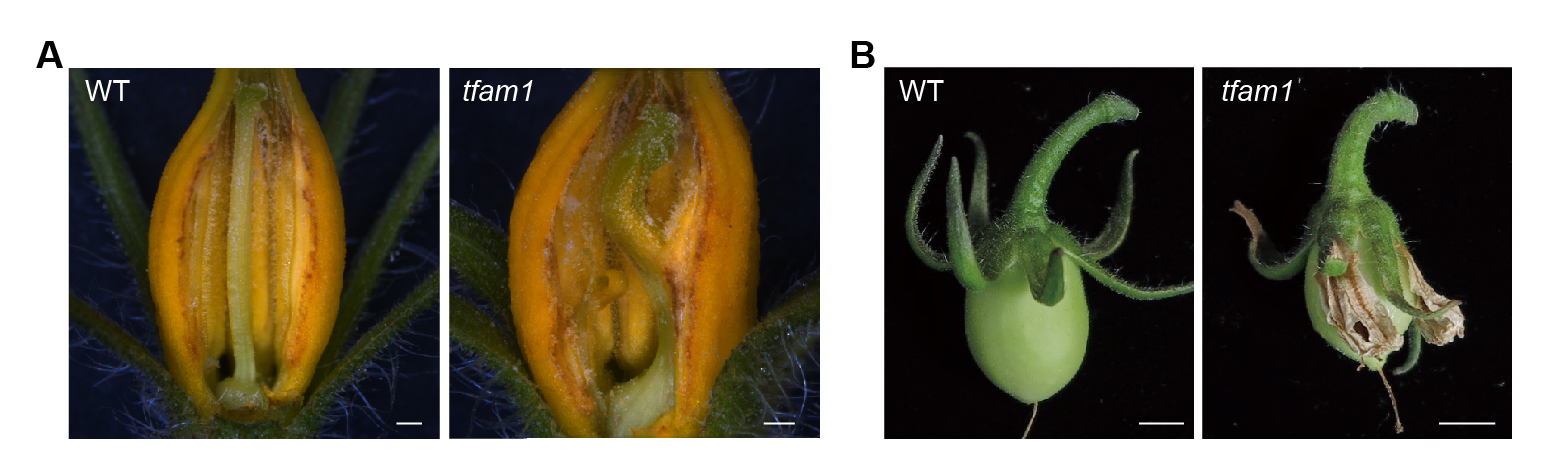


**Figure S9. *TFAM1* regulates floral organ development and abscission.** (A and B) Representative flowers (A) and fruits (B) of WT and *tfam1* single mutant. Scale bars, 500 μm (A), 0.5 cm (B).


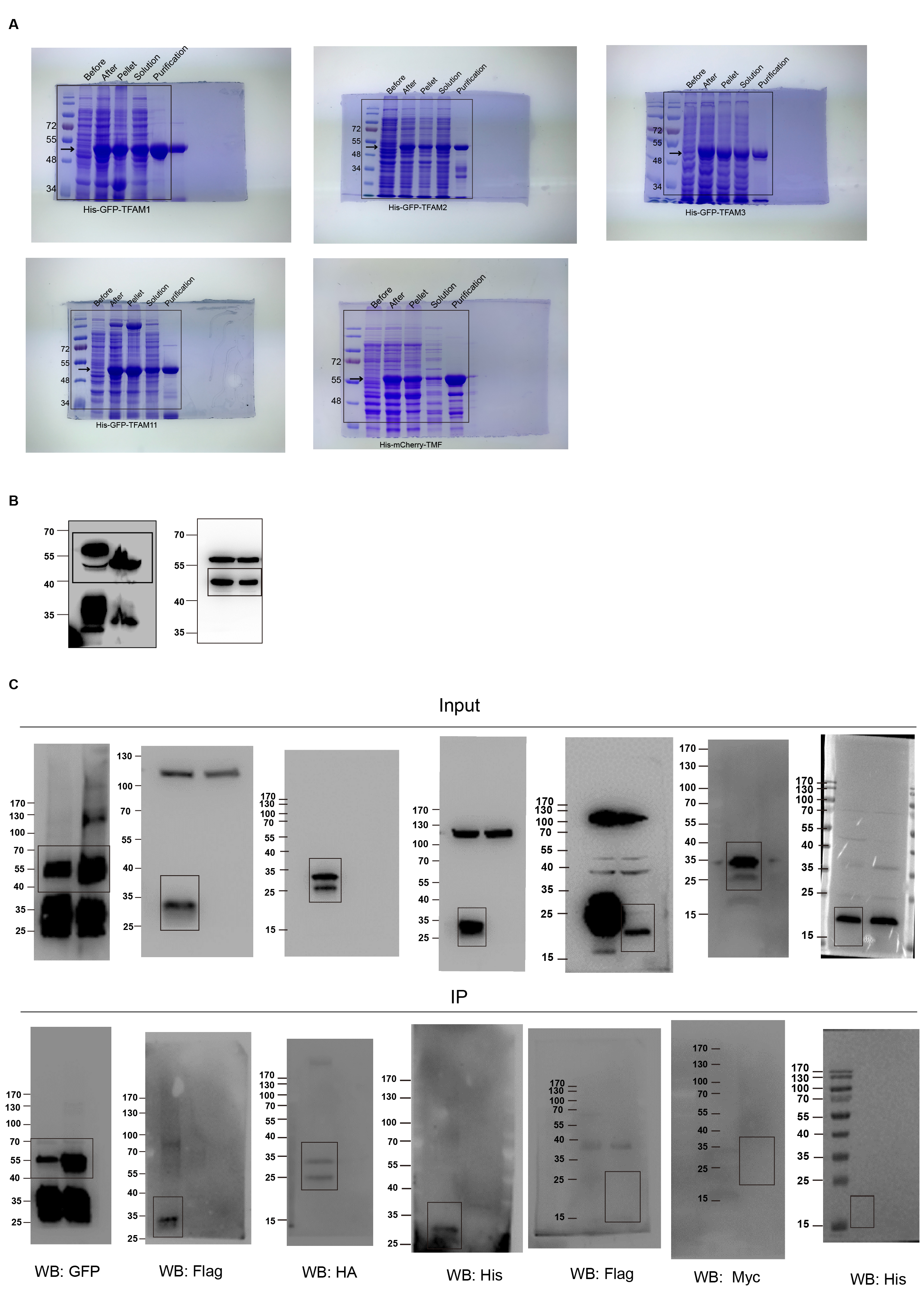


**Figure S10. Uncropped images for Western blot gel.** (A) Correspondence to Fig. S3B. (B). Correspondence to Fig. S7B. (C). Correspondence to Fig. S7C. The areas marked with black box are cropped into figures.
