# Supplementary material for: Heterotypic transcriptional condensates formed by prion-like paralogous proteins canalize flowering transition in tomato
Source: Genome Biol. 2022 Mar 14;23:78. doi: 10.1186/s13059-022-02646-6 (PMC8919559; doi:10.1186/s13059-022-02646-6)
Supplement: Supplementary file 8 — Additional file 8. Review history. [file 13059_2022_2646_MOESM8_ESM.docx]

Review History

**First round of review**

**Reviewer 1**

**Are you able to assess all statistics in the manuscript, including the appropriateness of statistical tests used?**

Yes, and I have assessed the statistics in my report.

**Comments to author:**

The manuscript by Huang et al. describes the paralogs of TMF, TMAFs function redundantly with TMF in controlling tomato inflorescence development. First, the authors comprehensively dissected the genetic relationship between TMF and TMAFs using different combinations of mutants. Additionally, they studied the phase separation of these transcription factors and addressed how do these transcriptional condensates repress AN expression. Last, they elegantly swapped the IDR regions between TFM and TFAMs to demonstrate the IDR is critical for their transcriptional repression abilities.

Although this genetic robustness mechanism in shoot meristem has been reported before (Rodriguez-Lea et al., Nature Genetics 2019; Yang et al., Nature Plants 2021), this study is still very significant and provides novel insights. Previous studies showed that genetic robustness is caused by upregulating the expression of 'backup players' in the background of ablation of the major players. However, to my knowledge, this study is the first to show that the genetic robustness in plant shoot meristem is caused by phase-separation-mediated protein interactions between paralogous proteins. I believe this finding will inspire other researchers in this field. I am also impressed by the amount of high-quality data that elegantly support the conclusions. I have a few concerns and minor suggestions.

1. In line 177, the authors claim that" … the unequal redundancy among these TFAMs is not only due to transcription level but also refers to protein level." First, I suggest the authors remove 'but also refers to protein level', except they have the western blot data to support this conclusion. Second, the expression level of a gene or its protein accumulation might not always correlate with the importance of this gene. This is particularly true for developmental genes, which are frequently expressed in a few cells at specific developmental stages. For instance, the CLV3 and other CLE genes, their expression level are extremely low, but ablation of these genes introduces striking phenotypes. So, in a gene family, the one with the highest expression might not always be the 'major player'. It would be nice if the authors discuss this point in the discussion part.

2. The authors performed pairwise interaction tests between TMF and three TFAMs and concluded that they form heterodimers. But I do not think it excludes the possibility that more than two proteins form an oligomer. They may all pack together. I think it is difficult to address this question using experiments, but at least the authors should discuss this possibility.

3. Line 144, "mutations accumulated in IDR regions", "mutations" should be changed to "variations".

4. The font size of the figures is too small. I think this is because most of the figures are busy, but there is still space to enlarge the letters.

5. In lines 270-271, '… generate a transgenic line carrying…'. I believe the authors must generate more than one line here, but just use one of them for this experiment. Please clarify this.

6. Figure S6 showed western blot data, but there this experiment was not described in the Materials and Methods part. The authors also did not provide the antibody information.

**Reviewer 2**

**Are you able to assess all statistics in the manuscript, including the appropriateness of statistical tests used?**

No, I do not feel adequately qualified to assess the statistics.

**Comments to author:**

The manuscript entitled "Heterotypic transcriptional condensates formed by prion-like paralogous proteins derived from gene duplication canalize flowering transition in tomato" by Huang et al. described the coordinated role of TMF and TFAMs in controlling the transcription of ANANTHA (AN) and stem cell fate. They discovered that TMF and TFAMs play unequal roles in controlling floral transition. In addition, they form different condensates due to the divergence in their IDR and demonstrated that have different capability in transcriptional regulation. They proposed that TMF and TFAMs form heterotypic transcriptional condensates in controlling flowering transition. The conclusion that TMF and TFAMs play unequal roles in controlling flowering transition and share inseparable relationship and synergistic effects in modulating flowering time and inflorescence architecture is quite convincing. They further discovered that TMF and TFAMs diverge in their IDRs and proposed that they play differential role in regulating floral transition related to their capability in forming heterotypical transcriptional condensates. Overall, it is an interesting story, which contains lots of data. But there are several concerns need to be addressed as below.

Major concerns:

1. The authors drew the conclusion that formation of heterotypic condensates is required for their functions heavily relies on the in vitro LLPS data showing that TMF shows the most remarkable compatibility with TFAM3 but barely fused with TFAM11 droplets. But this reviewer is not convinced whether the formation of heterotypic transcriptional condensates is required for TMF and TFAMs to perform function. To draw such a conclusion, the authors should provide direct experimental evidence that formation of heterotypic condensates is required for their function in transcriptional regulation and floral transition. For instance, the authors can address whether ALOG family transcription factors function more efficiently in repressing the transcription of AN when they form heterotypic condensates? Without further data, it is inappropriate to state that the formation of heterotypic condensates is required for its role in controlling floral transition. Based on current set of data, it is probably more appropriate to simply state that formation of distinct condensates is required for TMF and TFAMs to perform functions.

2. High quality intracellular localization data of TMF and TFAMs and their spatial association are very important to understand their functional coordination in SAM during floral transition. The authors should consider visualizing the localization of their fluorescent fusion proteins with their expression driven by their own promoter under their mutant background. Unfortunately, most of intracellular localization data were obtained in protoplast in this manuscript, which might cause some artifact.

3. The authors argued that transcriptional compensation does not exist, as the results showed that loss of TMF fails to cause transcriptional upregulation of TFAM1/2/3 for compensation. To draw such a conclusion, the authors should detect transcription of TFAM1/2/3 within certain cell types in SAM of TMF loss-of-function mutant, as the change in cell fate of those cells is crucial for floral transition. Therefore, the authors should employ alternative approach to detect the transcription of TFAMs in TMF loss-of-function mutant, enabling the detection of the transcription levels at single cell resolution.

4. The authors used GFP or mCherry fusion proteins to perform the in vitro LLPS experiments. In order to avoid the interference from the fusion of fluorescent protein, the authors might consider using proteins labeled with fluorescent dyes to see whether they can obtain similar results, especially for the results shown in Figure S4.

Minor concerns:

1. The authors stated that the phase separation capacity of TFAM2 is weaker than TFAM1 and TFAM3 since it is more sensitive to salt, which is probably an incorrect statement. Actually, increase in the sensitivity to salt does not necessarily mean that it has weak separation capability. The authors should consider rewording this statement.

2. Overall, the introduction section is too long; the authors should consider shortening it, which will increase the readability of this manuscript.

3. The authors stated that "This inefficient compatibility of TFAM11 protein with TMF and other TFAMs is consistent with its dispensable role in regulating flowering and inflorescence architecture, providing a molecular support from a protein behavior perspective.", which needs more evidence to support this statement. The prerequisite for this statement is that interaction between TMF and TFAMs is indeed absolutely required for their transcriptional activity.

4. Better to add a brief figure legend for Figure S5.

**Authors Response**

**Point-by-point responses to the reviewers’ comments:**

Reviewer #1:

The manuscript by Huang et al. describes the paralogs of TMF, TMAFs function redundantly with TMF in controlling tomato inflorescence development. First, the authors comprehensively dissected the genetic relationship between TMF and TMAFs using different combinations of mutants. Additionally, they studied the phase separation of these transcription factors and addressed how do these transcriptional condensates repress AN expression. Last, they elegantly swapped the IDR regions between TFM and TFAMs to demonstrate the IDR is critical for their transcriptional repression abilities.

Although this genetic robustness mechanism in shoot meristem has been reported before (Rodriguez-Lea et al., Nature Genetics 2019; Yang et al., Nature Plants 2021), this study is still very significant and provides novel insights. Previous studies showed that genetic robustness is caused by upregulating the expression of 'backup players' in the background of ablation of the major players. However, to my knowledge, this study is the first to show that the genetic robustness in plant shoot meristem is caused by phase-separation-mediated protein interactions between paralogous proteins. I believe this finding will inspire other researchers in this field. I am also impressed by the amount of high-quality data that elegantly support the conclusions. I have a few concerns and minor suggestions.

*Response: We appreciate the Reviewer’s positive comments on the novelty and broad impact of our study. We also appreciate and agree with the suggestions for improving our manuscript. Below, please find our responses to all questions and comments.*

1. In line 177, the authors claim that" … the unequal redundancy among these TFAMs is not only due to transcription level but also refers to protein level." First, I suggest the authors remove 'but also refers to protein level', except they have the western blot data to support this conclusion. Second, the expression level of a gene or its protein accumulation might not always correlate with the importance of this gene. This is particularly true for developmental genes, which are frequently expressed in a few cells at specific developmental stages. For instance, the CLV3 and other CLE genes, their expression level are extremely low, but ablation of these genes introduces striking phenotypes. So, in a gene family, the one with the highest expression might not always be the 'major player'. It would be nice if the authors discuss this point in the discussion part.

*Response: Thank the Reviewer for sharing the thoughtful insights and we completely agree with suggestions. We have modified the wording inthe revised text and discussed this point in the discussion part (Line 149-150, Page 6, Line 427-431, Page 17).*

2. The authors performed pairwise interaction tests between TMF and three TFAMs and concluded that they form heterodimers. But I do not think it excludes the possibility that more than two proteins form an oligomer. They may all pack together. I think it is difficult to address this question using experiments, but at least the authors should discuss this possibility.

*Response: We thank the Reviewer for bringing up this great point and appreciate the Reviewer for understanding the technical challenges to experimentally address this question. Based on genetic analysis using extensive combinations of TFAM mutants and pairwise interaction tests in tomato cells, one can reasonably expect that TMF and three TFAMs form an oligomer. However, all four proteins have typical disorder regions and the molecular size of four proteins is very close, which indeed challenge the efficient co-expression and distinguishability by immune-blotting. Strikingly, inspired and encouraged by the Reviewer’s comments, we have overcome the technical difficulties after several rounds of failures for co-expressing four proteins simultaneously in tobacco leaves and performed the Co-immunoprecipitation assay to test the existence of TFAM protein complex. Indeed, as the Reviewer commented, TMF and three TFAMs physically interacted to form a heterotypic protein complex. We have added this data to supplemental Fig. S7 and updated the wording in the revised text. (Line 349-352, Page 14).*

3. Line 144, "mutations accumulated in IDR regions", "mutations" should be changed to "variations".

*Response: That’s a good point. While this whole sentence has been removed in the revised manuscript for concentrating the introduction part, we have thoroughly checked the main text to ensure there is no any other case.*

4. The font size of the figures is too small. I think this is because most of the figures are busy, but there is still space to enlarge the letters.

*Response: Thanks for the advice. We have enlarged the font size and optimized the organization of Figures. We have divided the initial Fig.1 into two independent figures, Fig.1 and Fig.2, now the revised manuscript has 6 main figures and 9 supplemental figures.*

5. In lines 270-271, '… generate a transgenic line carrying…'. I believe the authors must generate more than one line here, but just use one of them for this experiment. Please clarify this.

*Response: We agree and have modified this sentence to improve clarity. (Line 246- 247, Page 10).*

6. Figure S6 showed western blot data, but there this experiment was not described in the Materials and Methods part. The authors also did not provide the antibody information.

*Response: Thanks for pointing this out. We have added relative information to the Methods part in the revised text (Line 513-522, Page 20).*

Reviewer #2:

The manuscript entitled "Heterotypic transcriptional condensates formed by prion-like paralogous proteins derived from gene duplication canalize flowering transition in tomato" by Huang et al. described the coordinated role of TMF and TFAMs in controlling the transcription of ANANTHA (AN) and stem cell fate. They discovered that TMF and TFAMs play unequal roles in controlling floral transition. In addition, they form different condensates due to the divergence in their IDR and demonstrated that have different capability in transcriptional regulation. They proposed that TMF and TFAMs form heterotypic transcriptional condensates in controlling flowering transition. The conclusion that TMF and TFAMs play unequal roles in controlling flowering transition and share inseparable relationship and synergistic effects in modulating flowering time and inflorescence architecture is quite convincing. They further discovered that TMF and TFAMs diverge in their IDRs and proposed that they play differential role in regulating floral transition related to their capability in forming heterotypical transcriptional condensates. Overall, it is an interesting story, which contains lots of data. But there are several concerns need to be addressed as below.

Major concerns:

1. The authors drew the conclusion that formation of heterotypic condensates is required for their functions heavily relies on the in vitro LLPS data showing that TMF shows the most remarkable compatibility with TFAM3 but barely fused with TFAM11 droplets. But this reviewer is not convinced whether the formation of heterotypic transcriptional condensates is required for TMF and TFAMs to perform function. To draw such a conclusion, the authors should provide direct experimental evidence that formation of heterotypic condensates is required for their function in transcriptional regulation and floral transition. For instance, the authors can address whether ALOG family transcription factors function more efficiently in repressing the transcription of AN when they form heterotypic condensates? Without further data, it is inappropriate to state that the formation of heterotypic condensates is required for its role in controlling floral transition. Based on current set of data, it is probably more appropriate to simply state that formation of distinct condensates is required for TMF and TFAMs to perform functions.

*Response: We thank the reviewer for raising this very insightful question and suggesting experiments to address it. Actually, we already have some data in Fig. 5 and supplemental Fig. S7 to support that the formation of heterotypic transcriptional condensates is required for TMF and TFAMs to function, where co-expression of TMF and TFAMs shows dramatically enhanced transcriptional repression on AN than TMF alone. In contrast, deletion of IDRs of TFAMs (loss of protein phase separation) fails to produce such effects. We regret not organizing the data and Figures well and not explaining them clearly in the main text.*

*We fully agree with the reviewer’s suggestion and have performed more experiments to substantiate the essentiality of formation of TMF-TFAM heterotypic condensates. First, given that the transcriptional activity assay is performed in living tobacco plants, we tested if the condensates formed by TMF-TFAMs visualize in the nucleus and if the condensation depends on their IDR domains in the same living plant system. As shown in Fig. 5 in revised manuscript, TMF interacts with TFAMs to form condensates and deletion of IDRs of TFAM proteins abolishes the condensation. Second, to demonstrate TMF and TFAMs indeed interact to form a protein complex, we performed the Co-immunoprecipitation assay using the same living plant system as suggested by Reviewer #1 (See details in Response to Reviewer #1 comments 2). The Co-IP assay showed that they indeed interact to form a protein complex and the protein complex formation relies on IDR domains. Third, we compared the transcriptional activity of TMF alone, TMF-TFAMs and TMF-truncated TFAMs without IDRs. The dual luciferase transcriptional activity assay showed that formation of TMF-TFAM transcriptional condensates substantially enhanced transcriptional repression on AN comparing to TMF alone. However, deletion of IDRs of TFAMs failed to formation of the transcriptional condensates and eliminated the enhancement of transcriptional repression, demonstrating the essentiality of formation of TMF-TFAM heterotypic condensates in transcriptional regulation.*

*We have added the data to the revised Fig. 5 and updated the wording in the revised manuscript (Line 346-354, Page 14).*

2. High quality intracellular localization data of TMF and TFAMs and their spatial association are very important to understand their functional coordination in SAM during floral transition. The authors should consider visualizing the localization of their fluorescent fusion proteins with their expression driven by their own promoter under their mutant background. Unfortunately, most of intracellular localization data were obtained in protoplast in this manuscript, which might cause some artifact.

*Response: Thank the reviewer for this valuable advice. We totally agree with the Reviewer that visualizing the localization of TFAM-GFP fusion proteins with their expression driven by their own promoters is ideal for analyzing condensates, which is particularly suitable for observing condensates formed by individual proteins in a plant system where production of transgenic lines does not need to experience long-time tissue culture, such Arabidopsis. However, the main focus of our study is on exploring heterotypic condensates formed by multiple interacted proteins. It is technically difficult and time-consuming to generate tomato transgenic lines harboring multiple fluorescent fusion proteins driven by their own promoters. To overcome this obstacle, we have optimized the tomato protoplast system and confirmed the stability of our system by comparing TMF condensates formed in tomato protoplast with those formed in living tomato plants driven expression by TMF native promoter [1]. We therefore adopted this protoplast system to facilitate visualization of heterotypic condensates formed by multiple interacted proteins at single cell resolution.*

*Nevertheless, we completely agree with the Reviewer that observing condensates formed by individual TFAM proteins in living tomato plants is important for excluding the possible artifact due to overexpression. To this end, we generated transgenic constructs driven by TFAM native promoters, including pTFAM3:TFAM3-GFP, pTFAM1:TFAM1-GFP and pTFAM2:TFAM2-GFP. Unfortunately, the progress of genetic transformation and tissue culture for the three constructs went differently. Only pTFAM3:TFAM3-GFP went smoothly and we have shown in Fig. 4 that TFAM3 forms condensates in nucleus in tomato shoot apical meristems, consistent with the scenario of tomato protoplasts.*

*We have just obtained several T0 seedlings for pTFAM1:TFAM1-GFP and pTFAM2:TFAM2-GFP. To observe their localization in shoot apical meristems, we have to cut the shoot apices from these precious T0 seedlings to dissect and screen the meristems for microcopy observation. Fortunately, after sacrificing almost all of the shoot apices of the T0 seedlings, we finally identified the meristems that express TFAM1-GFP and TFAM2-GFP fluorescence. They showed similar punctate localization as they did in tomato protoplast while the signals are not ideal might due to the unstable expression in T0 plants from tissue culture. These results have excluded the possibility that the TFAM condensates are due to protein over-expression (Response Fig. 1). Accordingly, we have added words to further clarify this point in the revised manuscript (Line 253-257, Page 10).*

*Response Fig. 1. Representative images showing the punctate localization of TFAM1 and TFAM2 driven by their own promoter in the nucleus of T0 transgenic plants. Scale bars, 5 µm.*

3. The authors argued that transcriptional compensation does not exist, as the results showed that loss of TMF fails to cause transcriptional upregulation of TFAM1/2/3 for compensation. To draw such a conclusion, the authors should detect transcription of TFAM1/2/3 within certain cell types in SAM of TMF loss-of-function mutant, as the change in cell fate of those cells is crucial for floral transition. Therefore, the authors should employ alternative approach to detect the transcription of TFAMs in TMF loss-of-function mutant, enabling the detection of the transcription levels at single cell resolution.

*Response: We agree with the reviewer that detection of transcription levels of TFAMs at single cell resolution is ideal for excluding the occurrence of compensation caused by transcriptional upregulation. Indeed, the single cell sequencing of different types of stem cells in tomato shoot apical meristem is the best way to address this concern, but it deserves a whole independent project with tremendous technical obstacles.*

*To address the reviewer’s concern, we have employed the only currently available single-cell resolution technique for transcripts detection: droplet digital PCR (ddPCR). ddPCR is a method for performing digital PCR that is based on water-oil emulsion droplet technology. The droplet digital PCR system partitions nucleic acid samples into thousands of nanoliter-sized droplets, and PCR amplification is carried out within each droplet, which enables the measurement of thousands of independent amplification events within a single sample (Response Fig. 2).*

*To perform the ddPCR, we carefully micro-dissected the fresh shoot apical meristems and prepared nanoliter-sized droplets at single-cell resolution for PCR. The results showed that loss of TMF does not cause up-regulation of any of three TFAM genes, consistent with the results of qRT-PCR (Fig. 4A and Additional file 1: Fig. S6). We have used the ddPCR results to replace the previous qRT-PCR data in the revised text and Figures.*

*Response Fig. 2. Droplet digital PCR assay showing transcription level of TFAM1/2/3 in WT and tmf mutant. (A) Schematic diagram showing the principle of the ddPCR system. (B) The ddPCR data showing the positive and negative droplets. Each positive droplet is assigned a value of 1, each negative droplet is assigned a value of 0 (zero).*

4. The authors used GFP or mCherry fusion proteins to perform the in vitro LLPS experiments. In order to avoid the interference from the fusion of fluorescent protein, the authors might consider using proteins labeled with fluorescent dyes to see whether they can obtain similar results, especially for the results shown in Figure S4.

*Response: Thanks for this comment. The GFP (488 nm) and mCherry (543 nm) have been extensively used as classical fluorescence proteins in both biology and chemistry fields for a long time because they can be individually detected even at low levels and present little spectral overlap when used together [2-4].*

*Indeed, labelling target proteins using appropriate fluorescent dyes is another option for monitoring behavior of multiple proteins when mixing together. However, the TMF and TFAM proteins have very high amino acid similarity and share many protein properties, it is almost impossible to find proper dyes that can distinguish these paralogous proteins well. We therefore respectively do not think fluorescent dyes are better choice than fluorescent proteins for this experiment.*

Minor concerns:

1. The authors stated that the phase separation capacity of TFAM2 is weaker than TFAM1 and TFAM3 since it is more sensitive to salt, which is probably an incorrect statement. Actually, increase in the sensitivity to salt does not necessarily mean that it has weak separation capability. The authors should consider rewording this statement.

*Response: We appreciate the Reviewer being thorough in evaluating our manuscript and reminding us to describe the results more properly. While sensitivity to salt can reflect phase separation property of a protein, we agree that one cannot simply claim that phase separation capacity of a protein merely relies on salt sensitivity. We have indeed used very mild words in the main text, as “It seems that the phase separation capacity of TFAM2 is weaker than TFAM1 and TFAM3 since it is more sensitive to salt”. Now, we have modified the words into “The phase separation of TFAM2 seems more sensitive to salt than that of TFAM1 and TFAM3, suggesting their distinct characteristics”. (Line 279-281, Page 11).*

2. Overall, the introduction section is too long; the authors should consider shortening it, which will increase the readability of this manuscript.

*Response: Thank the Reviewer for this advice. We have trimmed down the introduction part by removing about 400 words in the revised manuscript. Now, it becomes more concise.*

3. The authors stated that "This inefficient compatibility of TFAM11 protein with TMF and other TFAMs is consistent with its dispensable role in regulating flowering and inflorescence architecture, providing a molecular support from a protein behavior perspective.", which needs more evidence to support this statement. The prerequisite for this statement is that interaction between TMF and TFAMs is indeed absolutely required for their transcriptional activity.

*Response: We thank the Reviewer for bringing our attention to the essentiality of formation of TMF-TFAM heterotypic condensates again. We have provided extensive data to address this concern in response to Reviewer #2 comments 1.*

4. Better to add a brief figure legend for Figure S5.

*Response: We have added a figure legend to this supplemental Fig. Now, it is Fig. S6 in the revised manuscript.*

*References*

*1. Huang X, Chen S, Li W, Tang L, Zhang Y, Yang N, et al. ROS regulated reversible protein phase separation synchronizes plant flowering. Nat Chem Biol. 2021, 17:549-557.*

*2. Fang Y, Soares L, Bonini NM. Design and implementation of in vivo imaging of neural injury responses in the adult Drosophila wing. Nat Protoc. 2013, 8:810-819.*

*3. Chudakov DM, Matz MV, Lukyanov S, Lukyanov KA. Fluorescent proteins and their applications in imaging living cells and tissues. Physiol Rev. 2010, 90:1103-1163.*

*4. Shaner NC, Steinbach PA, Tsien RY. A guide to choosing fluorescent proteins. Nat Methods. 2005, 2:905-909.*

**Second round of review**

**Reviewer 1**

The authors have addressed all of my concerns. It is a compelling story!

**Reviewer 2**

This reviewer is pleased to see that the authors have addressed most of the concerns raised during last round of review. However, this reviewer still wants to see the co-condensation of different transcription factors in vitro using fluorescein labeled proteins rather than the proteins fused with GFP or mCherry. For instance, the authors could re-perform some of the experiments in this revised manuscript, e.g. Figure S5, using fluorescein labeled proteins. As pointed out by this reviewer in previous reviewer comments, this might cause artifact. If it is indeed the case, it might change the main conclusion the authors drew in this manuscript.

**Authors Response**

**Point-by-point responses to the reviewers’ comments:**

Reviewer 1#:

The authors have addressed all of my concerns. It is a compelling story!

*Response: We are glad to see that all the concerns have been addressed and appreciate the Reviewer’s positive comments on the novelty and broad impact of our study.*

Reviewer 2#:

This reviewer is pleased to see that the authors have addressed most of the concerns raised during last round of review. However, this reviewer still wants to see the co-condensation of different transcription factors in vitro using fluorescein labeled proteins rather than the proteins fused with GFP or mCherry. For instance, the authors could re-perform some of the experiments in this revised manuscript, e.g. Figure S5, using fluorescein labeled proteins. As pointed out by this reviewer in previous reviewer comments, this might cause artifact. If it is indeed the case, it might change the main conclusion the authors drew in this manuscript.

*Response:* *We are glad to see that our efforts have addressed most of the concerns. We thank the Reviewer for this additional comment and fully understand* *that* *the Reviewer raised this concern again. We regret not clarifying this well in our previous response to reviewer text, and also regret not explaining the point clearly in the main text.*

*Indeed, while fusion of distinct fluorescence proteins represents a reliable and the most widely used approach to test protein-protein co-localization or interactions, labelling proteins with fluorescent dyes also serves as an effective way to perform such assays, especially when fusion of fluorescence protein might interfere function of target proteins. The fact that stable transgenic expression of TMF/TFAM tagged with fluorescent protein can fully complement the phenotypes of mutant plants demonstrated that fusion of fluorescent protein did not interfere functions of TMF/TFAM. We therefore used fusion of fluorescent protein to facilitate real-time visualization of droplet interactions. Now, we have added according words in the main text to clarify this point.*
